# Active PD-L1 incorporation within HIV virions functionally impairs T follicular helper cells

**DOI:** 10.1371/journal.ppat.1010673

**Published:** 2022-07-05

**Authors:** Olivia Munoz, Riddhima Banga, Rachel Schelling, Francesco Andrea Procopio, Andrea Mastrangelo, Pauline Nortier, Khalid Ohmiti, Jean Daraspe, Matthias Cavassini, Craig Fenwick, Laurent Perez, Matthieu Perreau

**Affiliations:** 1 Services of Immunology and Allergy, Lausanne University Hospital, University of Lausanne, Lausanne, Switzerland; 2 Electron Microscopy Facility, University of Lausanne, Lausanne, Switzerland; 3 Services of Infectious Diseases, Lausanne University Hospital, University of Lausanne, Lausanne, Switzerland; Vaccine Research Center, UNITED STATES

## Abstract

The limited development of broadly neutralizing antibodies (BnAbs) during HIV infection is classically attributed to an inadequate B-cell help brought by functionally impaired T follicular helper (Tfh) cells. However, the determinants of Tfh-cell functional impairment and the signals contributing to this condition remain elusive. In the present study, we showed that PD-L1 is incorporated within HIV virions through an active mechanism involving p17 HIV matrix protein. We subsequently showed that in vitro produced PD-L1high but not PD-L1low HIV virions, significantly reduced Tfh-cell proliferation and IL-21 production, ultimately leading to a decreased of IgG1 secretion from GC B cells. Interestingly, Tfh-cell functions were fully restored in presence of anti-PD-L1/2 blocking mAbs treatment, demonstrating that the incorporated PD-L1 proteins were functionally active. Taken together, the present study unveils an immunovirological mechanism by which HIV specifically exploits the regulatory potential of PD-L1 to suppress the immune system during the course of HIV infection.

## Author summary

During HIV infection, the development of effective BnAbs remains a rare phenomenon, occurring in only 15–20% of HIV-infected individuals after years of infection. Although multiple mechanisms may be involved, recent studies have suggested that functional impairment of Tfh cells, through immune checkpoint (IC)/IC-Ligand (IC-L) interactions, may lead to a decrease in B-cell help leading to low BnAbs production. Our laboratory recently showed that PD-L1 was predominantly expressed on lymph node (LN) migratory dendritic cells located predominantly in extra-follicular areas, implying that the source of IC-L contributing to Tfh-cell functional impairment may be independent of cellular expression of IC-L. These observations prompted us to investigate the potential contribution of IC-L incorporated within HIV virion envelope to Tfh-cell functional impairment. We subsequently demonstrated that PD-L1 was incorporated into a large fraction of HIV virions in the plasma of viremic HIV-infected individuals. Interestingly, PD-L1 remains active when incorporated into HIV virions envelope and could impaired Tfh-cell proliferation, resulting in decreased IgG1 production by B cells in vitro. These findings demonstrate an unsuspected mechanism contributing to the regulation of Tfh-cell function, which may contribute to the low production of BnAbs by B cells during HIV infection.

## Introduction

Functional impairment of HIV-specific T cells also called “T-cell exhaustion” is considered to represent one of the major obstacles to achieve an immunological control of HIV infection and is currently attributed to inhibitory signals triggered by immune checkpoint molecules (ICs) [1–3].

Indeed, following T-cell receptor (TCR) engagement, activation or inhibition of T-cell responses depends upon the balance between stimulatory and inhibitory signals, on the type of molecules engaged or ligands involved, and the availability of signaling molecules [4–6]. IC molecules interact with one or several ligands expressed by one or various cell types [7]. Notably, the expression of IC-ligands (IC-Ls) is either inducible or constitutively expressed on antigen presenting cells (APCs) such as dendritic cells (DCs), macrophages, B cells or on a variety of non-hematopoietic cells [8]. In the LCMV model of chronic viral infection, both APCs and LCMV-infected cells express IC-ligands such as Programmed Death-Ligand 1 (PD-L1) [9]. PD-L1 expression is indeed rapidly upregulated on LCMV infected cell subsets comprising B and T lymphocytes, natural killer cells, DCs, macrophages, and granulocytes which contribute to inhibit TCR signaling of LCMV-specific T cells, therefore lowering their effector functions [9,10].

Consistent with chronic LCMV infection, T cells exhaustion occurs from the early phase of HIV infection [11]. Notably, HIV-specific CD4 and CD8 T cells co-express multiple ICs, which expression levels correlated with antigen load (HIV viremia) and disease progression and were associated with impaired proliferation capacity, cytokine production and/or cytotoxicity [1–3,12–20]. Interestingly, additional studies revealed that lymph node (LN) germinal center (GC) T follicular helper (Tfh) cells, co-expressing programmed cell death protein 1 (PD-1) and T cell immunoreceptor with Ig and ITIM domains (TIGIT), were functionally impaired and may provide an inadequate B-cell help during HIV infection [21–24]. Indeed, the generation of BnAbs occurs only in a limited fraction (<10%) of HIV-infected individuals and requires a minimum of 2 to 4 years of infection [25–27].

However, the origin of the negative signals leading to this phenomenon remain elusive. Indeed, in LNs, IC-Ls such as PD-L1, PD-L2 (PD-1 ligands) and CD155 (TIGIT ligand) are predominantly expressed on migratory DCs and macrophages located in the paracortex area and to a lower extent on GC B cells [23]. These observations indicate that the cellular expression of IC-Ls does not fully explain the functional impairment of Tfh cells, and suggest that the source of IC-Ls interfering with Tfh cell functionality might be independent on the cellular expression of IC-Ls.

Interestingly, recent studies in the cancer field have demonstrated that IC-Ls are incorporated within extracellular vesicles such as exosomes and that their interaction with IC-expressing CD8 T cells was sufficient to reduce CD8 T cell functionality [28,29]. These findings indicated that the inhibitory signals triggered by IC/IC-L interactions do not necessarily require cell-to-cell interactions and proposed that soluble IC-L levels could predict the tumor response to treatment and clinical outcome in cancer [28,30–32].

The incorporation of host molecules such as HLA-DR, LFA-1, ICAM-1 or α4β7 integrins within HIV virions is also a well-documented mechanism [33–45]. Notably, these incorporated molecules were proposed to create selective advantages during HIV transmission [37] and/or infection [40,41,44]. However, none of these studies focused on the potential incorporation of immunoregulatory molecules such as IC-Ls within HIV virions.

In the present study, we investigated the potential IC-L incorporation into exosomes and/or HIV virions during HIV infection and its impact on Tfh cell functions. We showed that HIV virions could incorporate functionally active PD-L1 and could therefore contribute to systemically suppress the immune system during the course of HIV infection.

## Results

### Plasmatic PD-L1 levels are increased in viremic HIV-infected individuals

**Fig 1:**
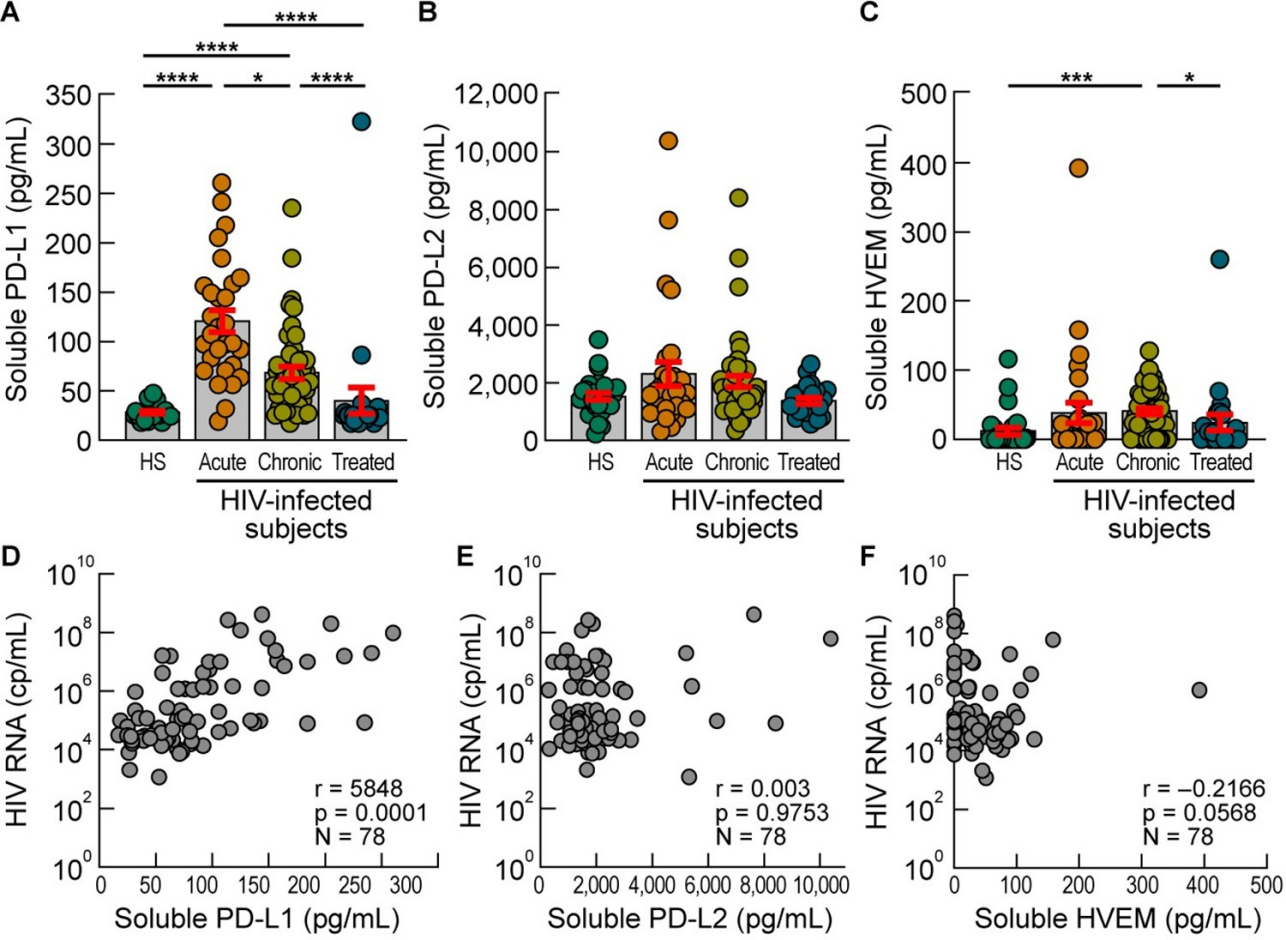
Plasmatic PD-L1 levels are increased in viremic HIV-infected individuals. **A-C.** Levels of plasmatic PD-L1 **(A)**, PD-L2 **(B)** and HVEM **(C)** of healthy donors (N = 28), viremic acute HIV-infected individuals (N = 29), viremic chronic HIV-infected individuals (N = 49) and ART-treated aviremic HIV-infected individuals (N = 23). **D-F**. Correlation between HIV viral load (copies/mL) and levels of PD-L1 **(D)**, PD-L2 **(E)** and HVEM **(F)** in plasma of viremic HIV-infected individuals (N = 86). Histograms correspond to the mean **(A-C)**, and red error bars correspond to the standard error of the mean (SEM) **(A-C)**. Black asterisks indicate statistical significance (* = *P*<0.05; *** = *P*<0.001; **** = *P*<0.0001). Statistical significance (*P* values) was obtained using one-way ANOVA (Kruskal-Wallis test) followed by a Dunn’s multiple comparison test **(A-C)** and by using Spearman’s rank correlations **(D-F)**.

The aim of the present study was to determine whether soluble IC-Ls might contribute to the T-cell functional impairment observed during the course of HIV infection. To achieve this objective, we comprehensively assessed the levels of soluble PD-L1, PD-L2 (ligands of PD-1) and HVEM (ligand of CD160) in plasma of untreated viremic HIV-infected individuals, during primary HIV infection (PHI), or during the chronic phase, or in plasma of treated aviremic chronically HIV-infected individuals. The cumulative data indicated that soluble PD-L1 levels were significantly increased in the plasma of viremic HIV-infected individuals during PHI and chronic HIV infection as compared to HIV uninfected or treated aviremic HIV infected individuals (*P*<0.0001) (Fig 1A). However, plasmatic levels of PD-L2 were not significantly different between the groups compared (*P*>0.05) (Fig 1B). Notably, plasmatic HVEM levels were significantly increased in the plasma of HIV-infected individuals during chronic HIV infection as compared to HIV uninfected individuals (*P*<0.001) or to treated aviremic HIV infected individuals (*P*<0.05) (Fig 1C). Interestingly, plasmatic PD-L1 levels directly correlated with HIV viremia (r = 0.5848; *P* = 0.0001) (Fig 1D), while no significant association was found between the plasmatic PD-L2 or HVEM levels and HIV viremia (*P*>0.05) (Fig 1E and 1F). Collectively, these data suggest that the quantitative changes of plasmatic PD-L1 levels are associated with HIV viral load.

### PD-L1^+^gp120/41^+^ extracellular vesicles represent the major source of PD-L1^+^ extracellular vesicles in plasma of viremic HIV-infected individuals

Since host cell molecules can be either incorporated into exosomes or HIV virions during the production processes [46,47], we conducted a series of experiments to determine the origin of PD-L1^+^ extracellular vesicles *i*.*e*. exosomal *versus* viral origins. To address this issue, PD-L1^+^ extracellular vesicles were captured from plasma of viremic HIV-infected or uninfected individuals using beads coated with anti-PD-L1 monoclonal antibodies (mAbs) (S1A Fig). Captured PD-L1^+^ extracellular vesicles were further characterized by isotope labelled mAbs targeting CD9, CD63 and CD81, markers of exosomes [48], and mAbs specific to gp120 or gp41 (gp120/41), proteins of HIV-1 virions by mass cytometry (S1A Fig). As control, isotype-control coated beads were also used (S1A Fig). Notably, minimal exosomes or HIV virions were captured by isotype-control coated beads as compared to anti-PD-L1 mAbs coated beads (*P*<0.001) (S1B and S1C Fig), demonstrating the specificity of anti-PD-L1 mAbs coated beads.

**Fig 2:**
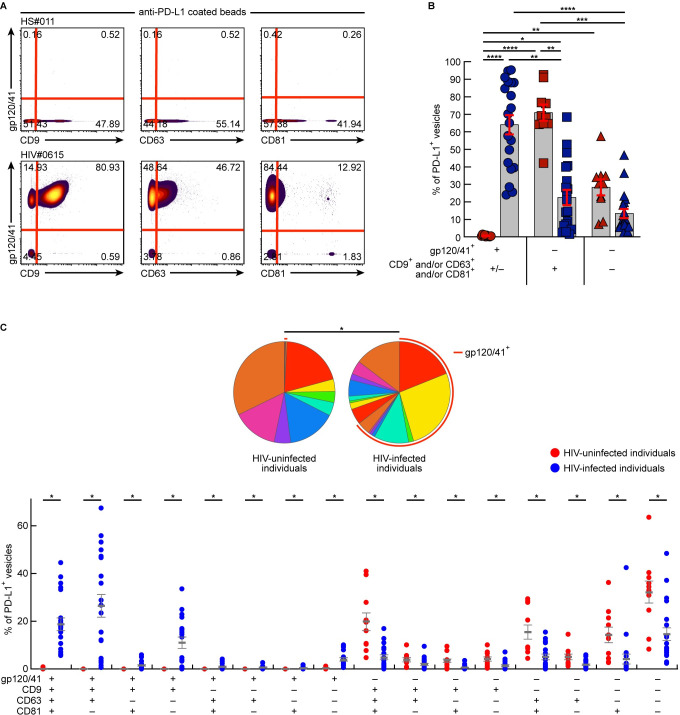
PD-L1 is incorporated within HIV virions and represent the major source of PD-L1+ extracellular vesicles in plasma of viremic HIV-infected individuals. Polystyrene beads coated with anti-PD-L1 mAbs were used to capture PD-L1^+^ vesicles in plasma from HIV-uninfected individuals (N = 10) and viremic HIV-infected individuals (N = 20). **A**. Representative mass cytometry profile of a plasma from one HIV-uninfected individual (HS#011) and one viremic HIV-infected individual (HIV#0615) captured by anti-PD-L1 mAbs coated beads and showing the incorporation level of gp120/41, CD9, CD63 and CD81 using isotope-labeled antibodies. **B.** Cumulative data showing the proportion of PD-L1^+^ vesicles with gp120/41 and/or CD9/CD63/CD81. **C.** Incorporated protein profiles of PD-L1^+^ extracellular vesicles captured from plasma of HIV-uninfected individuals (N = 10) or viremic HIV-infected individuals (N = 20). All the possible combinations of PD-L1^+^ vesicle with gp120/41, CD9, CD63 and CD81 are shown on the x-axis and the percentage of PD-L1^+^ vesicles harboring specific markers combination is shown on the y-axis. The pie chart summarizes the data, and each slice corresponds to the proportion of PD-L1^+^ vesicles. Histograms correspond to the mean and error bars correspond to the SEM **(B-C)**. Black stars indicate statistical significance (* = *P*<0.05; **; *P*<0.01, ***, *P*<0.001, **** = *P*<0.0001). Red arc corresponds to PD-L1^+^ vesicles harboring gp120/41 (**c**). Statistical significance (*P* values) was obtained using one-way ANOVA (Kruskal-Wallis test) followed by a Dunn’s multiple comparison test **(B)** or using Mann Whitney test **(C)**. Statistical analysis of the global phenotype of PD-L1^+^ vesicles (pie charts) **(C)** was performed by partial permutation tests using the SPICE software as previously described [92].

The incorporated protein profiles of PD-L1^+^ extracellular vesicles captured from plasma of HIV-uninfected individuals and viremic HIV-infected individuals are shown in Fig 2. The representative and cumulative data indicated that the incorporated protein profile of plasmatic PD-L1^+^ extracellular vesicles of viremic HIV-infected individuals was significantly different from the one of HIV-uninfected individuals (*P*<0.0001) (Fig 2A and 2C). Interestingly, about 64% of PD-L1^+^ extracellular vesicles captured from plasma of viremic HIV-infected individuals incorporated gp120/41, while unspecific gp120/41 staining was not detected (<1%) on PD-L1^+^ extracellular vesicles captured from plasma of HIV-uninfected individuals (*P*<0.0001) (Fig 2A and 2B). In contrast, the proportion of PD-L1^+^ extracellular vesicles harboring markers of exosomes in absence of gp120/41 was significantly increased in HIV-uninfected individuals as compared to viremic HIV-infected individuals (71% *versus* 22.5%, *P*<0.01) (Fig 2A and 2B). Of note, no significant differences in the proportion of PD-L1^+^ extracellular vesicles harboring markers of exosomes or virions were observed between plasma of HIV-infected individuals collected during PHI and chronic HIV infection (*P*>0.05) (S2 Fig). In depth analysis revealed that PD-L1^+^ extracellular vesicles captured from plasma of viremic HIV-infected individuals were significantly enriched in gp120/41^+^CD9^+^CD63^+^CD81^-^, gp120/41^+^CD9^+^CD63^+^CD81^+^ and gp120/41^+^CD9^+^CD63^-^CD81^-^ vesicles as compared to HIV-uninfected individuals (*P*<0.05) (Fig 2C). In contrast, PD-L1^+^ extracellular vesicles captured from plasma of HIV-uninfected individuals were significantly enriched in gp120/41^-^CD9^+^CD63^+^CD81^+^, gp120/41^-^CD9^-^CD63^+^CD81^+^ and gp120/41^-^CD9^-^CD63^-^CD81^+^ vesicles as compared to viremic HIV-infected individuals (*P*<0.05) (Fig 2C). These data indicate that PD-L1^+^ gp120/41^+^ extracellular vesicles represent the major source of PD-L1^+^ extracellular vesicles in plasma of viremic HIV-infected individuals.

### PD-L1^+^ HIV virions represent a major fraction of plasmatic virions of viremic HIV-infected individuals

**Fig 3:**
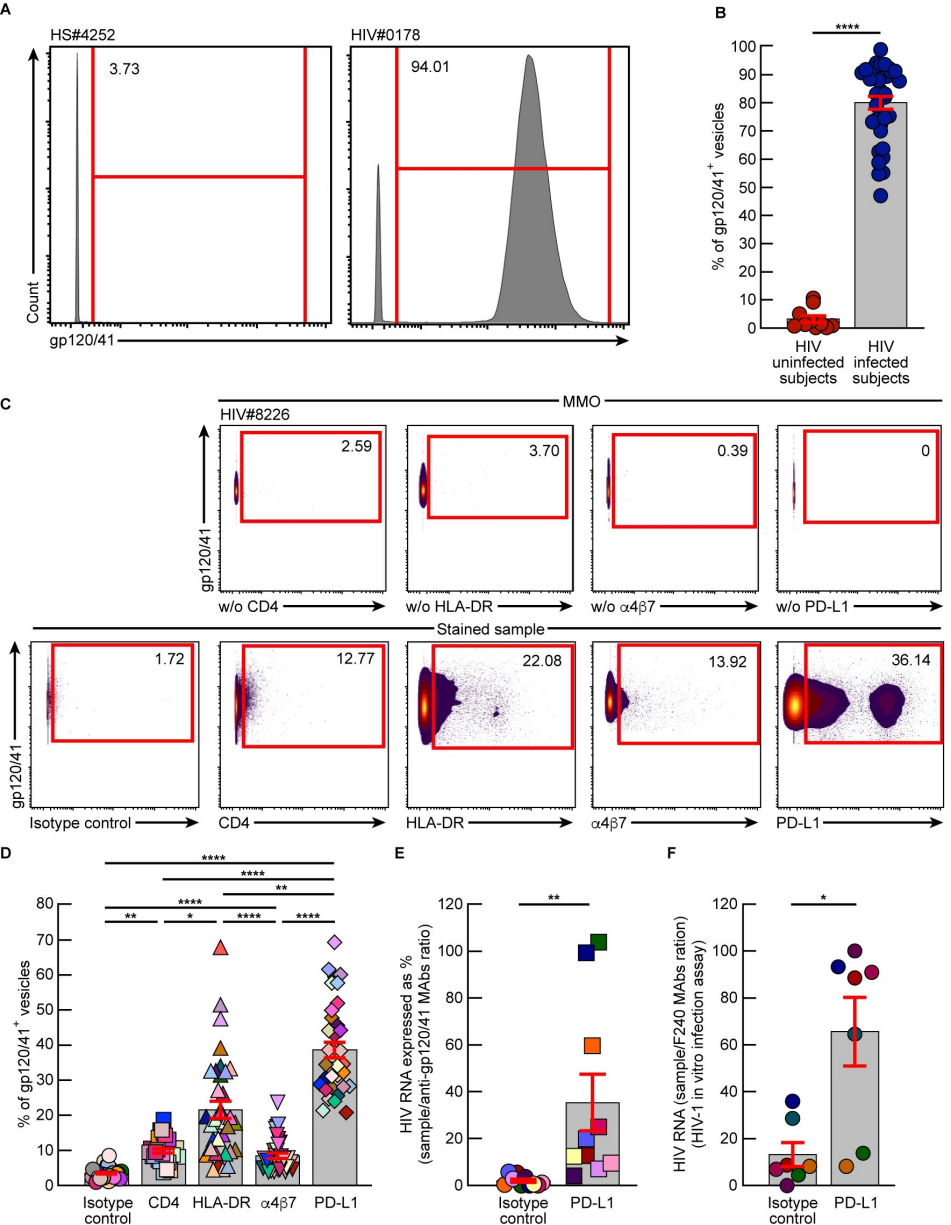
PD-L1^+^ HIV virions represent a major fraction of plasmatic virions of viremic HIV-infected individuals. Polystyrene beads coated with a pool of anti-gp120/41 mAbs were used to capture HIV virions from plasma of viremic HIV-infected individuals (N = 37). **A.** Representative histogram of mass cytometry profile of gp120/41^+^ vesicles from plasma of one HIV-uninfected individual (HS#4252) and one viremic HIV-infected individual (HIV#0178). **B.** Proportion of beads with captured gp120/41^+^ vesicles of plasma from HIV-uninfected individuals (N = 10) and viremic HIV-infected individuals (N = 37). **C.** Representative mass cytometry profile of plasmatic gp120/41^+^ vesicles captured from one HIV-infected individual (HIV#8226). **D.** Cumulative data mass cytometry profiles of plasmatic gp120/41^+^ vesicles captured from HIV-infected individuals (N = 37). **E.** Proportion of gp120/41^+^ vesicles captured with beads coated with anti-PD-L1 mAbs or isotype-control as compared to beads coated with the pool of anti-gp120/41 mAbs (considered as reference) from plasma of viremic HIV-infected individual (N = 10). **F.** Proportion of HIV RNA levels detected in culture supernatants of activated CD4 T cells exposed to vesicles captured using beads coated with anti-PD-L1 mAbs or an isotype-control as compared to beads coated with anti-gp41 F240 mAbs (considered as reference) from plasma of viremic HIV-infected individual (N = 7). MMO; mass-minus one staining. Histograms correspond to the mean and red error bars correspond to SEM **(B, D-F)**. Black stars indicate statistical significance (* = *P*<0.05; ** = *P*<0.01; **** = *P*<0.0001). Statistical significance (*P* values) was obtained using Mann Whitney test **(B, E-F)** or using one-way ANOVA (Friedman test) followed by a Dunn’s multiple comparison test **(D)**.

We next determined the proportion of plasmatic virions harboring PD-L1 in plasma of viremic HIV-infected individuals directly *ex vivo*. To address this question, HIV virions were captured from plasma of viremic HIV-infected using anti-gp120/41 mAbs coated beads (S3 Fig). As control, plasma from HIV-uninfected individuals were also used. Notably, minimal capture of vesicles was observed in HIV-uninfected individuals (*P*<0.0001), demonstrating the specificity of anti-gp120/41 mAbs coated beads to capture HIV virions (Fig 3A and 3B). Captured HIV virions from viremic HIV-infected individuals were further characterized using isotope labelled anti-PD-L1 mAbs. The incorporation of HLA-DR, known to be incorporated [37,40,49] and CD4, known to be poorly incorporated [34] were also used as internal controls. The positivity of each marker was defined by a mass-minus one (MMO) strategy as previously described [50] (Fig 3C). The representative and cumulative data indicated that HLA-DR and PD-L1 were significantly more frequently detected on HIV virions than CD4 and α4β7 (*P*<0.05) (Fig 3C and 3D). Interestingly, PD-L1 incorporation within plasmatic virions was significantly more frequently detected (about 38% of virions) than HLA-DR (*P*<0.01), demonstrating the preponderance of PD-L1 incorporation into HIV virions *in vivo* (Fig 3C and 3D).

To confirm that PD-L1^+^gp120/41^+^ vesicles corresponded to HIV-1 virions, PD-L1^+^ extracellular vesicles were captured from plasma of viremic HIV-infected individuals using anti-PD-L1 mAbs coated magnetic beads and assessed for the presence of HIV-1 RNA levels directly *ex vivo* as previously described [23]. Notably, isotype-control coated beads were used as controls and HIV virions were also captured using anti-gp120/41 mAbs coated beads and were considered as reference. The cumulative data indicated that HIV RNA levels detected in plasmatic PD-L1^+^ extracellular vesicles were significantly higher as compared to isotype-control coated beads (*P*<0.01) and corresponded to about 35% of the one detected in anti-gp120/41 mAbs coated beads, confirming the mass cytometry data (Fig 3E).

Finally, in order to determine whether PD-L1^+^ extracellular vesicles were infectious, we performed an *in vitro* HIV-1 infection assay. For this purpose, PD-L1^+^ extracellular vesicles were captured from plasma of viremic HIV-infected individuals using anti-PD-L1 mAbs coated magnetic beads and were used to inoculate pre-activated CD4 T cells isolated from HIV-uninfected individuals as previously described [51]. As internal control, HIV virions were also captured using non-neutralizing anti-gp41 mAbs coated beads. Finally, plasma samples were also incubated with isotype-control coated beads as controls. Culture supernatants were collected at day 0 and 14 and assessed for the presence of HIV-1 RNA. After 14 days of culture, HIV RNA levels were significantly higher in culture supernatants of CD4 T cells exposed to PD-L1^+^ extracellular vesicles as compared to cells exposed to isotype-control coated beads (*P*<0.05), and correspond to about 60% of the RNA levels detected when virions were captured using anti-gp41 mAbs coated beads (Fig 3F).

Taken together, these data indicate that plasmatic PD-L1^+^ HIV virions are infectious *in vitro* and represent a major fraction of plasmatic virions of viremic HIV-infected individuals.

### PD-L1^+^ virions can be produced from PD-L1^+^ CD4 T cells or monocyte-derived macrophages *in vitro*

**Fig 4:**
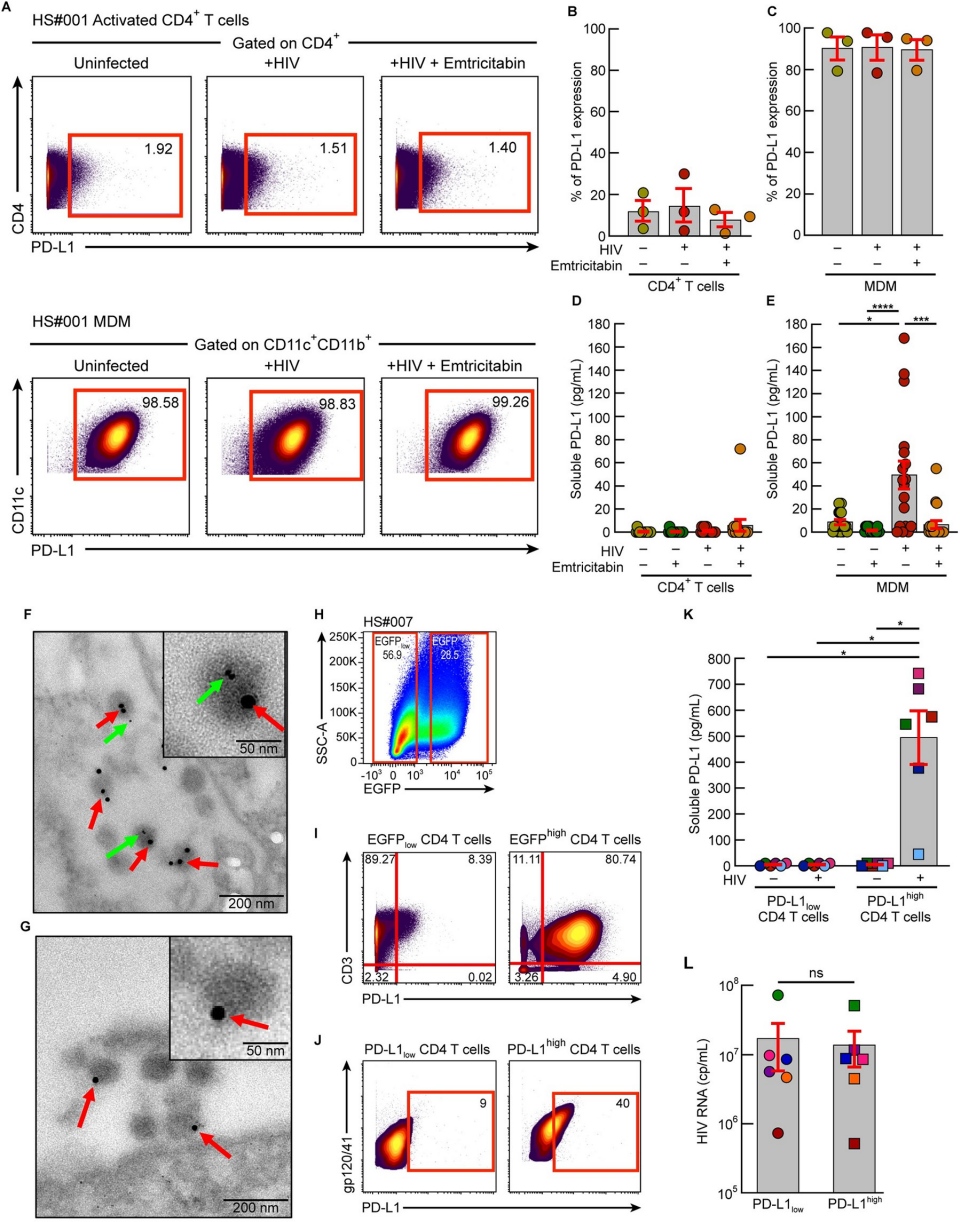
PD-L1^+^ virions can be produced from PD-L1^+^ CD4 T cells or monocyte-derived macrophages *in vitro*. Activated CD4 T cells or MDM from HIV-uninfected individuals (N = 7) were exposed or not to HIV lab-derived variants in presence or in absence of emtricitabin. At day 10 post-infection, the proportion of cells expressing PD-L1 was assessed by mass cytometry and the soluble PD-L1 levels in the supernatants were assessed by ELISA. **A.** Representative mass cytometry profiles showing PD-L1 expression on activated CD4 T cells or MDM (HS#001). **B,C** Cumulative data showing the proportion of activated CD4 T cells (**B**) or MDM (**C**) expressing PD-L1. **D,E** Cumulative data showing the levels of soluble PD-L1 in the supernatants activated CD4 T cells (**D**) or MDM (**E**). **F,G** Representative electron microscopy pictures HIV-infected MDM using a dual-size immunogold labeling stained with (**F**) rabbit anti-p24 antibodies and revealed by gold-labelled (15 nm) anti-rabbit Abs and biotinylated anti-PD-L1 mAbs revealed by gold-labelled (6 nm) streptavidin or (**G**) rabbit anti-p24 antibodies revealed by gold-labelled (15 nm) anti-rabbit Abs and gold-labelled streptavidin (6 nm). Representative image of 10 different images obtained from HIV-infected MDM **H**. Gating strategy of sorted EGFP/PD-L1 transduced CD4 T cells. Activated CD4 T cells isolated from HIV-uninfected individuals were transduced with PD-L1/EGFP-lentivirus. EGFP_low_ and EGFP^high^ CD4 T cells were sorted using FACS Aria. **I.** Representative mass cytometry profile showing PD-L1 expression on EGFP_low_ or EGFP^high^ transduced CD4 T cells. **J**. Representative mass cytometry profile showing PD-L1 incorporation within HIV virions captured in culture supernatants of PD-L1_low_ or PD-L1^high^ transduced CD4 T cells. **K**. Soluble PD-L1 levels assessed in day 14 culture supernatants of PD-L1_low_ or PD-L1^high^ transduced CD4 T cells. **L**. HIV RNA levels assessed in day 14 culture supernatants of HIV lab-derived variant-infected PD-L1_low_ or PD-L1^high^ transduced CD4 T cells. Histograms correspond to the mean and error bars correspond to SEM **(B-E, K)**. Black asterisks indicate statistical significance (* = *P*<0.05; *** = *P*<0.001; **** = *P*<0.0001). n.s. corresponds to not significant (**L**). Statistical significance (*P* values) was obtained using one-way ANOVA (Friedman test) followed by a Dunn’s multiple comparison test **(B-E, K)**.

PD-L1 is constitutively expressed on macrophages or dendritic cell populations and might be induced on HIV-infected CD4 T cells [52]. We therefore determined the role of HIV infection on PD-L1 expression and consequently in the release of PD-L1^+^ HIV virions. To address this issue, PD-L1 expression levels were first assessed on activated CD4 T cells or monocyte-derived macrophages (MDM), isolated/derived from HIV-uninfected individuals, exposed or not to replication competent lab-derived HIV variants by mass cytometry. Notably, cell cultures were treated or not with emtricitabin to prevent HIV replication. The representative mass cytometry examples and cumulative data indicated that MDM expressed higher levels of PD-L1 as compared to CD4 T cells, independently of HIV infection or replication (Fig 4A–4C). Interestingly, HIV infection did not significantly influence PD-L1 expression on CD4 T cells or MDM *in vitro* (*P*>0.05) (Fig 4A–4C).

We subsequently assessed the influence of HIV-infection on soluble PD-L1 levels in culture supernatants (Fig 4D and 4E). The cumulative data indicated that the soluble PD-L1 levels were significantly increased in the culture supernatants of MDM exposed to replication competent HIV virus in absence of emtricitabin (*P*<0.05) (Fig 4E), suggesting that PD-L1^+^ virions could be produced from MDM *in vitro*. Of note, HIV RNA levels detected in culture supernatants of HIV-infected MDM were not significantly different from HIV-infected CD4 T cells (*P*>0.05) (S4 Fig).

To further confirm the incorporation of PD-L1 into HIV virions, HIV particles were visualized in HIV-infected MDM using a dual-size immunogold labeling assay by electron microscopy as previously described [53]. Briefly, sections of HIV-infected MDM were stained with 1) rabbit anti-p24 antibodies (directed against HIV capsid protein) and revealed by gold-labelled (15 nm) anti-rabbit Abs and 2) with biotinylated anti-PD-L1 mAbs, revealed by gold-labelled (6 nm) streptavidin (Fig 4F). Notably, the degree of unspecific binding of gold-labelled streptavidin was assessed in sections stained with rabbit anti-p24 antibodies, gold-labelled anti-rabbit Abs and gold-labelled streptavidin (Fig 4G). The representative electron microscopy pictures indicated that PD-L1 was detected on p24-labelled particles (Fig 4F), demonstrating its incorporation into HIV viral particles. Notably, the examination of multiple sections of HIV-infected MDMs showed frequent p24 gold-labelled viruses with extremely rare unspecific detection of gold-labelled streptavidin (Fig 4G), demonstrating the specificity of anti-PD-L1 labeling.

Incorporation of host proteins involves several mechanisms that may depend on the cell lineage [46,54]. To determine whether PD-L1 incorporation into HIV virions could occur from CD4 T cells expressing higher levels of PD-L1, activated CD4 T cells isolated from HIV-uninfected individuals were transduced with PD-L1/EGFP-lentivirus. Transduced cells, expressing high or low PD-L1 levels, were sorted on the basis of EGFP expression and exposed to replication competent lab-derived HIV variants for 14 days (Fig 4H). Notably, an EGFP-lentivirus lacking PD-L1 reporter gene was also used as control. Levels of PD-L1 expression on EGFP_low_ and EGFP^high^ CD4 T cells were assessed by mass cytometry and confirmed a higher PD-L1 expression on EGFP/PD-L1 transduced CD4 T cells as compared to EGFP_low_ cells (Fig 4I). In addition, PD-L1 incorporation into *in vitro* produced HIV virions was assessed by mass cytometry using gp120/41 mAbs coated beads. The representative mass cytometry profile indicated that about 40% of HIV virions incorporated PD-L1 when produced from HIV-infected PD-L1^high^ CD4 T cells (Fig 4J). Culture supernatants were collected and soluble PD-L1 levels were assessed. The cumulative data indicated that the soluble PD-L1 levels were significantly increased in the culture supernatants of HIV-infected PD-L1^high^ CD4 T cells as compared to HIV-infected PD-L1_low_ CD4 T cells (*P*<0.05) (Fig 4K). Notably, HIV RNA levels detected in culture supernatants of HIV-infected PD-L1_low_ were not significantly different from HIV-infected PD-L1^high^ CD4 T cells (*P*>0.05) (Fig 4L).

Taken together, these data indicate that PD-L1^+^ HIV virions could be produced from HIV-infected PD-L1^+^ CD4 T cells or MDM *in vitro*.

### PD-L1 incorporation into HIV virions involves direct interactions between p17 HIV matrix proteins and the intracytoplasmic domain of PD-L1

**Fig 5:**
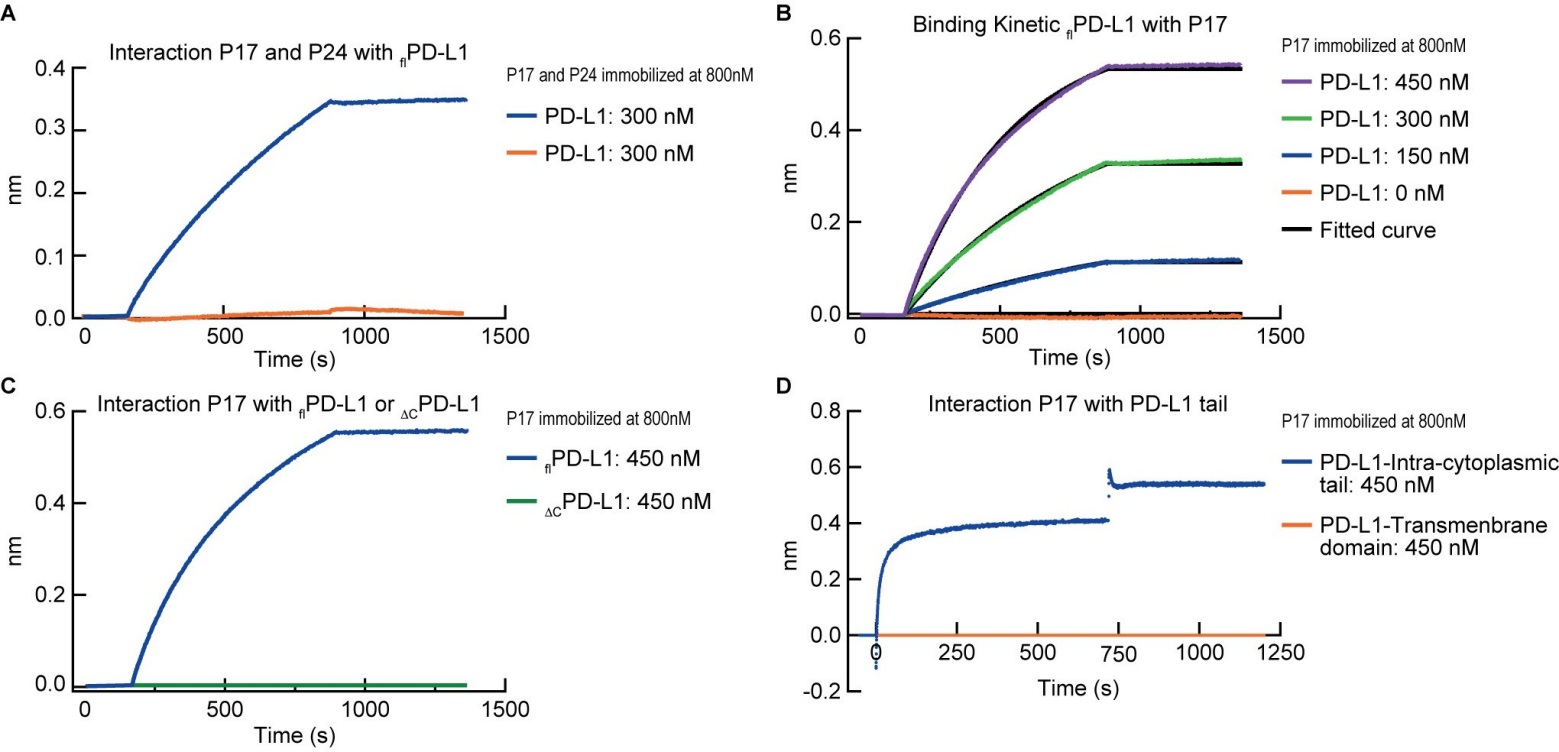
PD-L1 incorporation into HIV virions involves direct interactions between p17 HIV matrix proteins and the intracytoplasmic domain of PD-L1. **A.** Full-length PD-L1 (_fl_PD-L1) proteins interactions with p17 (blue curve) or p24 (orange curve) HIV proteins determined by BLI. **B**. Binding kinetic of _fl_PD-L1 proteins interactions with p17 HIV proteins at different concentrations. Fitting with the 1:1 model is shown in black. **C**. p17 HIV proteins interactions with _fl_PD-L1 (blue curve) or _ΔC_PD-L1 (green curve) proteins. **D.** The interaction of the transmembrane domain peptide (orange curve) and intracytoplasmic tail peptide (blue curve) of PD-L1 with p17 were determined by BLI. BLI sensorgrams are representative examples of experiments repeated three times.

During the budding process, p17 HIV matrix proteins play a major role in the membrane targeting of p24 HIV capsid proteins and envelope glycoproteins and may contribute to the host protein incorporation into HIV virions [55]. In this context, we conducted a series of *in vitro* experiments to determine whether PD-L1 incorporation into HIV virions may implicate an active phenomenon involving interactions with HIV matrix or capsid proteins. To address this issue, we first investigated the potential interactions between recombinant full-length PD-L1 protein (Phe19-Thr290; _fl_PD-L1) with immobilized recombinant p17 or p24 proteins using biolayer interferometry (BLI) based assay (Fig 5A). The data demonstrated that p17 protein interacted with _fl_PD-L1 protein, while no interaction was detected between p24 protein and recombinant _fl_PD-L1 protein, demonstrating the specificity of p17/_fl_PD-L1 interactions (Fig 5A).

To further determine the affinity of p17/_fl_PD-L1 interactions, p17 was immobilized and exposed to various concentrations of _fl_PD-L1 ranging from 0 to 450 nM (Fig 5B). The data demonstrated that p17 protein interacted with _fl_PD-L1 protein with a constant of dissociation (KD) value of 0.3 10^−9^ M (±2.10^−11^) (Fig 5B).

Finally, to identify the specific PDL1 domain interacting with p17, we repeated the aforementioned experimental strategy and compared the capacity of immobilized p17 protein to interact with either 1) recombinant PD-L1 proteins lacking the intracellular C-terminal tail (Phe19-Thr239; _ΔC_PD-L1), 2) the transmembrane domain peptide of PD-L1 (Thr239—Phe259) or 3) the intracytoplasmic tail peptide of PD-L1 (Arg260-Thr290) (Fig 5C and 5D). The interaction between immobilized p17 protein and _fl_PD-L1 were also assessed as control (Fig 5C). The data indicated that immobilized p17 protein did not interact with _ΔC_PD-L1 protein (Fig 5C) or with the peptide covering the transmembrane domain of PD-L1 (Fig 5D), while the interactions between p17 and peptide covering the intracytoplasmic tail of PD-L1 were conserved (Fig 5D). These data demonstrated that p17 HIV matrix protein specifically interacted with the intracytoplasmic tail of PD-L1 with a high affinity.

Taken together, these data support an active recruitment of PD-L1 with HIV virion envelop through a process involving at least in part direct interactions between p17 HIV matrix protein and the intracytoplasmic domain of PD-L1.

### PD-L1^+^ HIV virions are functionally active and contribute to Tfh cell functional impairment *in vitro*

To determine whether PD-L1 molecules remained active when incorporated into HIV virion envelope, three distinct approaches were used.

First, we evaluated the influence of *in vitro* produced PD-L1^high^ *versus* PD-L1_low_ HIV virions on TCR signaling. To address this issue, we first used a PD-1^+^ Jurkat T cell line modified to express the luciferase gene under the control of NFAT response elements [56]. Briefly, PD-1^+^ Jurkat T cells were stimulated with anti-CD3/CD28 mAbs for 24 hours in presence or in absence of increasing doses of *in vitro* produced PD-L1^high^ HIV virions that were immobilized using non-neutralizing anti-gp41 mAbs. As controls, cells were stimulated with anti-CD3/CD28 mAbs in presence of anti-gp41 mAbs or remained unstimulated. Stimulated cells were also exposed to immobilized recombinant PD-L1 proteins and PD-L1_low_ HIV virions as controls. In some conditions, cells were also cultured with blocking anti-PD-L1/2 mAbs.

**Fig 6:**
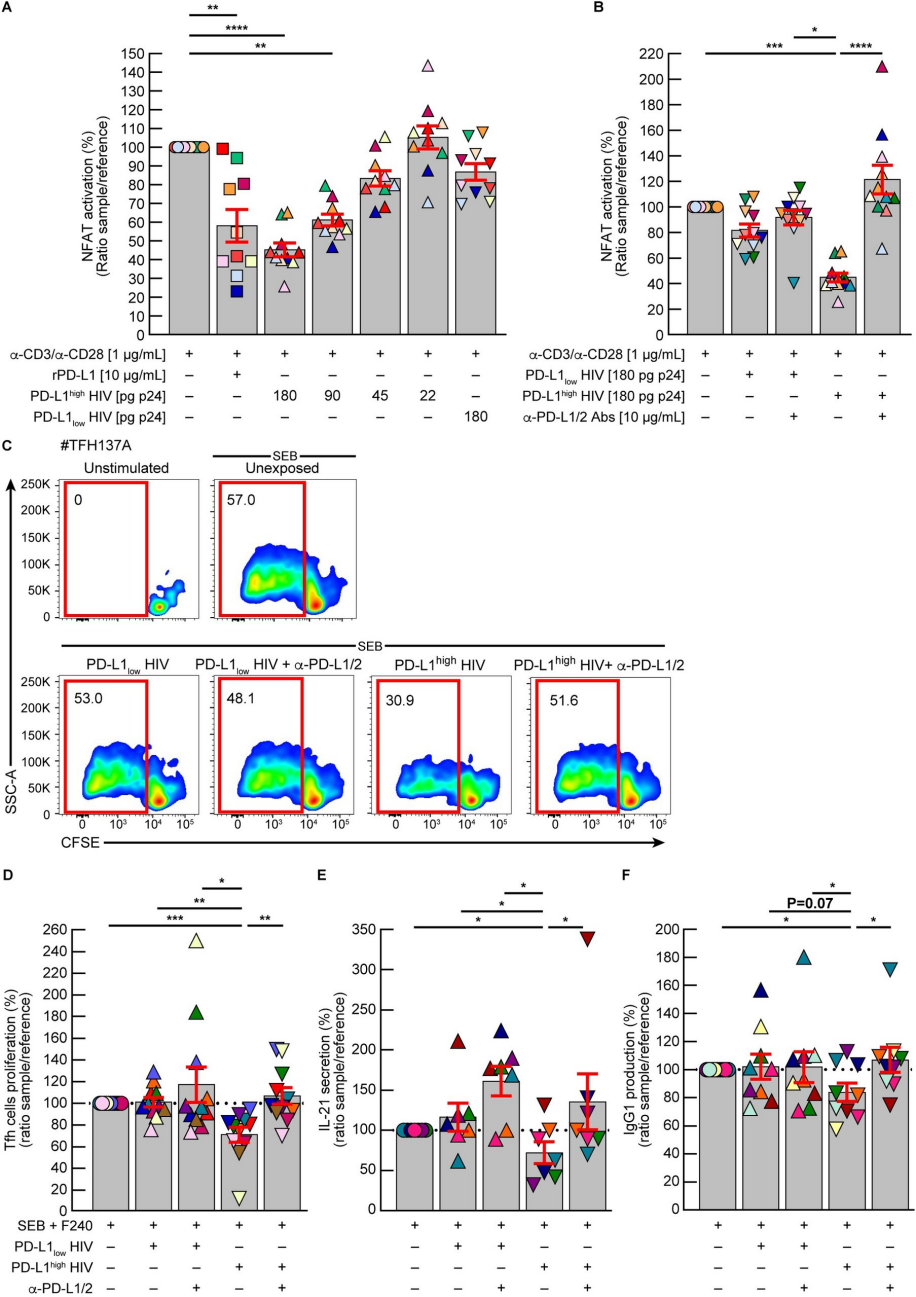
PD-L1^+^ HIV virions are functionally active and contribute to Tfh cell functional impairment *in vitro*. PD-1/NFAT reporter Jurkat cells were stimulated with anti-CD3/CD28 mAbs and cultured in presence of recombinant PD-L1 protein (rPD-L1), PDL1^high^ or PD-L1_low_ HIV virions. Immobilization of HIV virions was performed using non-neutralizing anti-gp41 F240 mAbs. **A.** Cumulative data showing the proportion of NFAT activation in presence of rPD-L1, *in vitro* produced PDL1^high^ HIV virions at various concentrations or PD-L1_low_ HIV virions as compared to cells stimulated with anti-CD3/CD28 mAbs in presence of anti-gp41 F240 mAbs (considered as reference) (N = 10). **B.** Cumulative data showing the proportion of NFAT activation in the presence of *in vitro* produced PD-L1_low_ or PDL1^high^ HIV virions in presence or absence of blocking anti-PD-L1/2 mAbs as compared to cells stimulated with anti-CD3/CD28 mAbs in presence of anti-gp41 F240 mAbs (considered as reference) (N = 11). Tfh cells isolated from tonsils of HIV-uninfected individuals (N = 10) were CFSE labelled and co-cultured with autologous GC B cells in presence of emtricitabin. Cells were stimulated with SEB in presence or absence of immobilized *in vitro* produced PD-L1^high^ or PD-L1_low_ HIV virions in presence or absence of blocking anti-PD-L1/2 mAbs. Immobilization of HIV virions was performed using non-neutralizing anti-gp41 F240 mAbs **C**. Representative flow cytometry profile of Tfh cell proliferation from one representative individual (TFH#137A). Cumulative data showing the proportion of **(D)** proliferating Tfh cells (CFSE_low_) (N = 10), **(E)** secretion of soluble IL-21 (N = 7) and **(F)** production of IgG1 (N = 9) in each condition as compared to Tfh cells stimulated with SEB in presence of anti-gp41 F240 mAbs (considered as reference). Histograms correspond to the mean and red error bars correspond to SEM (**A, B, D-F**). Black stars indicate statistical significance (* = *P*<0.05; ** = *P*<0.01; *** = *P*<0.001, **** = *P*<0.0001). Statistical significance (*P* values) was obtained using one-way ANOVA (Friedman test) followed by a Dunn’s multiple comparison test (**A, B, D**) or using one-way Mann Whitney test (**E-F**).

The cumulative data indicated that PD-L1^high^ HIV virions, but not PD-L1_low_ HIV virions, significantly reduced TCR-mediated NFAT activation in a dose dependent manner (*P*<0.01) (Fig 6A), that was fully restored in presence of blocking anti-PD-L1/2 mAbs treatment (Fig 6B).

We next assess the capacity of *in vitro* produced PD-L1^high^ *versus* PD-L1_low_ HIV virions to interfere with TCR signaling on tonsillar CD4 T cells. Briefly, CD4 T cells were isolated from tonsils of HIV-uninfected individuals and stimulated with anti-CD3/CD28 mAbs for 5 minutes in presence or in absence of *in vitro* produced PD-L1_low_ HIV virions or PD-L1^high^ HIV virions. As controls, cells remained unstimulated. The phosphorylation of ZAP70 and SLP76 was assessed by mass cytometry on PD-1^-^ and PD-1^+^ memory CD4 T cells and used as markers of early TCR signaling cascade. The representative and cumulative data indicated that PD-L1^high^ HIV virions reduced SLP76 phosphorylation in PD-1^+^ memory CD4 T cells (*P*>0.05) (S5 Fig). Notably, the phosphorylation of ZAP70 remained unchanged in all conditions tested. Taken together, these data suggested that PD-L1^+^ HIV virions may engage PD-1 molecules on memory CD4 T cells and may interfere with TCR signaling.

We finally investigated whether PD-L1^+^ HIV virions may interfere with Tfh cell functionality. To address this issue, Tfh cells were isolated from tonsils of HIV-uninfected individuals, CFSE labelled, co-cultured with autologous GC B cells and stimulated with *Staphylococcus* enterotoxin B (SEB) in presence or in absence of *in vitro* produced PD-L1^high^ or PD-L1_low_ HIV virions. Notably, to ensure the specific influence of *in vitro* produced HIV virions, PD-L1^high^ or PD-L1_low_ HIV virions were immobilized on wells using non-neutralizing anti-gp41 F240 mAbs. In some conditions, cells were also cultured with mAbs that blocked the interactions between PD-1 and PD-L1/2. As controls, stimulated cells were also exposed to non-neutralizing anti-gp41 mAbs used to immobilize HIV virions. Tfh cell functionality was assessed using direct (proliferation capacity and IL-21 secretion) and indirect (IgG1 production from GC B cells) assays.

The representative and cumulative data indicated that PD-L1^high^ HIV virions, but not PD-L1_low_ HIV virions, significantly reduced Tfh cell proliferation (Fig 6C and 6D), IL-21 secretion (Fig 6E) and IgG1 production from GC B cells (Fig 6F) that were fully restored in presence of blocking anti-PD-L1/2 mAb treatment (*P*<0.05) (Fig 6C–6F). Taken together, these data demonstrate that PD-L1^+^ HIV virions were functionally active and could mediate T-cell functional impairment *in vitro*.

## Discussion

The development of BnAbs is dependent on the emergence of mutations not only in the CDR3 regions but more surprisingly in the framework regions of Abs [57,58]. These mutations are selected during repeated affinity maturation processes requiring efficient GC responses, which are mediated by appropriate B-cell help from Tfh cells [59]. The actual paradigm indicate that appropriate GC B cell co-stimulation is brought by well positioned Tfh cells in appropriate numbers and involves the expression of membrane costimulatory molecules such as CD40-L and SLAM and soluble molecules such as IL-4, IL-21 and CXCL13[60]. However, the limited occurrence of BnAbs in HIV-infected individuals suggested an inadequate support from Tfh cells [25–27].

In this context, several studies revealed that LN GC Tfh cells, co-expressing ICs such as PD-1 and TIGIT, were functionally impaired during HIV infection [21–24]. However, in LNs, PD-1 or TIGIT ligands *i*.*e*. PD-L1, PD-L2 and CD155 are predominantly expressed on cells locating in extrafollicular area [23], suggesting that the source of IC-Ls interfering with Tfh cell functionality might be independent on the cellular expression of IC-Ls.

Interestingly, recent observations made in the field of oncology revealed that IC-ligand proteins either cleaved by matrix metalloproteinases (MMPs) into soluble IC-L fragments [61,62] or incorporated into membrane-bound extracellular vesicles such as exosomes [28,29] were functionally active and were sufficient to reduce CD8 T cell functions *in vitro* [28,29,63]. These studies improved the understanding of the mechanisms involved in the regulation of the immune response and provided the rational for a better understanding of IC-L-mediated immune evasion during the course of HIV infection.

Interestingly, membrane bound IC-Ls and soluble PD-L1 levels are also increased during chronic HIV infection and several studies proposed to use such levels as potential markers of HIV-1 persistence, virological failure, persistent activation, exhaustion and/or predictors of viral rebound [64].

In this context, we investigated whether soluble IC-L levels might be increased during HIV infection in various virological settings *i*.*e*. during primary HIV infection, during chronic infection, or in plasma of treated aviremic chronically HIV-infected individuals. Consistent with previous studies [61,64], we found that the plasmatic PD-L1 levels were significantly increased in the plasma of HIV-infected individuals during primary and chronic HIV infection as compared to HIV uninfected or treated aviremic HIV infected individuals. Interestingly, plasmatic PD-L1 levels directly correlated with HIV viremia, suggesting that plasmatic PD-L1 quantitative changes might be associated with HIV viral load. However, this phenomenon was not observed for plasmatic PD-L2 and HVEM levels were only significantly increased in plasma of viremic chronically HIV-infected individuals. The fact that soluble IC-Ls might be produced by distinct cell lineages or regulated by distinct mechanisms might have contributed to these differences.

Since HIV virions can incorporate host cell molecules during the production process [33–45], we therefore conducted a series of experiments to determine the origin of PD-L1^+^ vesicles (exosomal *versus* viral origins). Consistent with previous studies [65], exosomes were characterized by the membrane incorporation of CD9, CD63 and/or CD81 in absence of gp120/41 (marker of viral origin). Since CD9, CD63 and/or CD81 were host molecules known to be incorporated to HIV-1 virons [66,67], the potential viral origin of PD-L1^+^ vesicles was considered in presence of gp120/41 incorporation, independently of the presence of CD9, CD63 and/or CD81. We found that plasmatic PD-L1^+^ vesicles incorporated gp120/41, contained HIV RNA and were able to *de novo* infect activated CD4 T cells, thus demonstrating that PD-L1 might be incorporated within HIV virions during the production process.

Incorporation of host proteins involves several mechanisms that may depend on the cell lineage (CD4 *versus* macrophage) [46,54], the abundancy of host molecules on the cell membrane, the potential specific interaction between viral and host proteins. Indeed, abundant host molecules (*e*.*g*. CD44) can be incorporated at the level of the lipidic envelope of the producing cell *via* a passive mechanism during the physiological viral budding process [68,69]. In addition, host molecules can also be incorporated *via* an active mechanisms involving the remodeling of the lipid composition of HIV membrane (*e*.*g*. CD59 [36,70] or the interaction of the host molecules (*e*.*g*. ICAM-1) with the matrix domain of gag polyprotein [71]. These selectively acquired functionally active molecules are considered to create selective advantages for HIV transmission and/or infection [44,45,72–74]. Finally, HIV virions may also use the endocytic pathway of macrophages that allows the virus to be taken within multivesicular endosomes [46]. These non-mutually exclusive mechanisms could contribute to explain the differences observed in the proteomic profiles of virions produced from T cells and macrophages *in vitro* [75].

A reconstructive approach demonstrated that PD-L1 incorporation could occur on virions produced from *in vitro* MDM or PD-L1 transduced CD4 T cells, demonstrating that PD-L1 incorporation within HIV virions did not depend on the cell lineage. We then explored whether PD-L1 incorporation within HIV virions might involved an active mechanism. We showed using a BLI based assay that p17 HIV matrix proteins interacted specifically with _fl_PD-L1 proteins with a KD value of 0.3 10^−9^ M. Additional investigation revealed that p17 proteins specifically interacted with the intracytoplasmic tail of PD-L1. These data support that the enrichment of PD-L1 within HIV virions is mediated at least in part by an active mechanism involving the interaction between p17 HIV matrix protein and the intracytoplasmic tail of PD-L1.

In contrast to IC-Ls expressed at the cell surface, soluble IC-Ls can diffuse in various body compartments *via* the blood and lymphatic circulation and exert an inhibitory effects by interacting with cell surface receptors either in the local micro-environment or distally. Notably, in the past few years, several studies were performed to trace exosome biodistribution *in vivo* in mouse models [76–78]. To address this issue, radio or fluorescently labeled exosomes were injected into the tail vein [76], the lymphatic route [76], intradermally [77] or subcutaneously [78] and exosome anatomical distribution was assessed using positron emission tomography (PET) or *in vivo* imaging systems, respectively. These studies showed that labeled exosomes accumulated in the spleen, the kidneys and in draining LNs [76–78].

The efficiency of IC-L^+^ exosomes to interfere with TCR-mediated signals is difficult to evaluate and would probably depend on the number of IC-L molecules incorporated into the exosomes and/or the exosome concentration in a specific micro-environment. However, using a cell line model, Poggio *et al*., estimated that the efficiency of PD-L1^+^ exosomes to interfere with IL-2 secretion from PD-1^+^ Jurkat cells did not differ from the one obtained with cell-to-cell interactions [79].

One of the key consequences of PD-L1 incorporation within HIV virions might rely on the potential accumulation of IC-L bearing virions at specific sites, physiologically deprived of natural IC-L expression, such as in GCs. Indeed, in LNs, IC-Ls such as PD-L1, PD-L2 or CD155 are mainly expressed on migratory dendritic cells that locate in extrafollicular area, while follicular dendritic cells (FDCs) and GC B cells poorly expressed IC-Ls [23]. However, HIV virions forming immune complexes with antibodies or complement molecules accumulate on FDC network [80,81]. Therefore, the accumulation of PD-L1^+^ virions in GC area might represent a major source of PD-L1, which may disturb HIV-specific Tfh cell positioning and functions [82].

To determine the consequences of such an event on Tfh cell functionality, we performed a series of assays to determine whether *in vitro* produced PD-L1^high^ HIV virions may interfere with TCR-mediated signaling. First, we used a PD-1^+^ Jurkat T cell line that was modified to express the luciferase gene under the control of NFAT response elements [56]. This first series of experiments showed that PD-L1^high^ HIV virions, but not PD-L1_low_ HIV virions, significantly reduced TCR-mediated NFAT activation in a dose dependent manner, that were fully restored in presence of blocking anti-PD-L1/2 mAbs treatment. Next, we assessed the influence of PD-L1^high^ HIV virions on early TCR mediated signaling and showed that PD-L1^high^ HIV virions substantially reduced SLP76 phosphorylation in PD-1^+^ memory CD4 T cells.

We subsequently focused on the influence of PD-L1^high^ HIV virions on primary Tfh cells. To address this issue, the TCR induced Tfh cell proliferation capacity was assessed in presence or in absence of immobilized *in vitro* produced PD-L1^high^ or PD-L1_low_ HIV virions. We showed that PD-L1^high^ HIV virions, but not PD-L1_low_ HIV virions, significantly reduced Tfh cell proliferation and IL-21 production, which translated into reduced IgG1 production from GC B cells. Interestingly, Tfh cell functions were fully restored in presence of anti-PD-L1/2 blocking mAbs treatment, demonstrating that the incorporated PD-L1 proteins were functionally active. The consequences of such an impairment on the qualitative profile of GC reaction would however require future investigations.

These findings indicated that the inhibitory signals triggered by IC/IC-L interactions do not necessarily require cell-to-cell interactions and echoed the observations made by the group of Chen *et al*., that recently demonstrated that purified PD-L1^+^ exosomes were able to reduce melanoma-specific CD8 T cell functions [28].

Taken together, the present study unveiled a mechanism by which HIV specifically exploits the regulatory potential of soluble IC-L molecules to systemically suppress the immune system during the course of HIV infection.

## Materials and methods

### Ethics statement

A total of 127 plasma collected from HIV infected patients were studied in the present study. Primary HIV infection was defined by an HIV viral load exceeding 10^5^ copies/mL and a negative immunodot. Chronic HIV infection was defined by an HIV viral load exceeding 10^3^ copies/mL for more than a year. All HIV plasma samples were collected in a retrospective study, approved by the ethical committee (Commission cantonale d’éthique de la recherche sur l’être humain; CER-VD; #2018–00847). Samples were all anonymized. As controls, 32 plasma were collected from HIV-negative individuals. The exclusion criteria were sign of acute or chronic viral hepatitis (HAV, HBV, HCV and HEV), prior diagnosis of autoimmune disease (*e*.*g*. rheumatoid arthritis, psoriasis, SLE), prior diagnosis of primary or secondary immunodeficiency (*e*.*g*. HIV infection) and current or past (last 4 weeks) use of medications that are known modify the immune response. Blood mononuclear cells were collected from 10 HIV-1 uninfected individuals. Tonsil mononuclear cells were collected from 10 HIV-1 uninfected individuals in prospective studies approved by the CER-VD (#PB-2016-02436) and all subjects provided a written informed consent. Mononuclear cells were isolated as previously described [83]. No statistical method was used to predetermine sample size. The sample size was estimated based on a previously published study [44]. Plasma and cells collected from HIV-uninfected individuals were collected in prospective studies approved by the CER-VD (#2018–01932) and all subjects provided a written informed consent.

### Antibodies

The following monoclonal antibodies (mAbs) were used for flow cytometry analysis (*c*.*f*. CD4 T-cell proliferation section): BUV737-conjugated anti-CD3 (UCHT1), BUV805-conjugated anti-CD4 (SK3), APC-H7-conjugated anti-CD19 (HIB19), and APC-conjugated anti-CD38 (HIT2) were purchased from Becton Dickinson. Pacific Blue-conjugated anti-PD-1 (EH12.2H7) was purchased from Biolegend. The following monoclonal antibodies (mAbs) were used for Tfh and GC B cell sorting experiments: ECD-conjugated anti-CD4 (SK3), PeCy7-conjugated anti-PD-1 (MIH1), PE-conjugated anti-IgD (IA6-2), APC-conjugated anti-CD38 (HIT2), APC-H7-conjugated anti-CD19 (HIB19). All abs were purchased from Becton Dickinson. The following antibodies were used for mass cytometry experiments: 115In-conjugated anti-CD4 (RPA-T4), 145Nd-conjugated anti-CD81 (5A6), 148Sm-conjugated anti-PD-L1 (29E.2A3), 150Nd-conjugated anti-CD63 (H5C6), 164Dy-conjugated isotype-control (MGI-45), 171Yb-conjugated anti-CD9 (HI9a), 173Yb-conjugated anti-α4β7 (vedolizumab; Takeda), 174Yb-conjugated anti-HLA-DR (L243), 197Au-conjugated Streptavidin (Sigma-Aldrich; #S9059), Antibodies against CD4, were purchased from Biolegend and were conjugated with Maxpar X8 Antibody Labeling Kit (Fluidigm/DVS). All other antibodies were purchased from Fluidigm/DVS. The following antibodies were used for mass cytometry experiments to assess the influence of PD-L1^+^ virions on early TCR mediated signaling of primary CD4 T cells. 89Y-conjugated anti-CD45 (HI30), 113In-conjugated anti-CD8a (RPA-T8), 115In-conjugated anti-CD4 (RPA-T4), 141Pr-conjugated anti-CD45 (HI30), 151Eu-conjugated anti-PD1 (EH12.2H7), 154Sm-conjugated anti-CD3 (UCHT1), 156Gd-conjugated anti-pSLP76 (pY128), 170Er-conjugated anti-CD45RA (HI100), 171Yb-conjugated anti-pZAP70 (17A), 173Yb-conjugated anti-CD45RO (UCHL1), 194Pt-conjugated anti-CD45 (HI30), 195Pt-conjugated anti-CD45 (HI30), 196Pt-conjugated anti-CD45 (HI30), 198Pt-conjugated anti-CD45 (HI30). Antibodies against CD8a, CD4, PD-1, CD45RO, and CD45 were purchased from Biolegend and were conjugated with Maxpar X8 Antibody Labeling Kit (Fluidigm/DVS). All other antibodies were purchased from Fluidigm/DVS.

### Quantification of soluble PD-L1, PD-L2, HVEM, IL-21 and IgG1 in culture supernatants and patients’ plasma

Quantification of soluble PD-L1 in culture supernatants and patients’ plasma was performed using ELISA (R & D Systems; #DB7H10) as previously described [84]. Quantification of soluble PD-L2, HVEM, IL-21 and IgG1 in culture supernatants and patients’ plasma was performed using luminex assay (Thermofisher).

### Characterization of PD-L1^+^ vesicles

PD-L1^+^ vesicles were characterized using mass cytometry based assay. Briefly, CBA Functional Beads (Becton Dickinson; Becton Dickinson; #558578, #558656, #560038, #558582 and #558586) were conjugated with either anti-PD-L1 mAbs (MIH1, Invitrogen; #14-5983-82) or an isotype-control (Biolegend; #401402) using the Functional Bead Conjugation Buffer Set protocol (BD; #558556) following the manufacturer’s instructions. Plasma were then incubated (2 hours, 4°C) with conjugated beads (9x10^4^ beads), washed (900g, 4 min) with PBS and incubated (30 min, 4°C) with a mix of biotinylated anti-gp120/41 mAbs (clones 10–1074, PG9, F240, 10E8, from NIH AIDS reagent program #12477, #12149, #7623, #12294; VRC07 (kind gift from G. Pantaleo; Lausanne University Hospital, Lausanne, Switzerland); 1μg/condition). Beads were then washed (900g, 4 min) with PBS, incubated (30 min at 4°C) with isotope labelled mAbs directed to PD-L1, CD9, CD63 and CD81 and fixed (1 hour, RT) in 3.2% PFA buffer. Labeled samples were acquired on a Helios instrument (Fluidigm) using a flow rate of 0.030 ml/min. Data were analyzed using Fluidigm Cytobank software package (Cytobank, Mountain View, CA). The proportion of PD-L1^+^ gp120/41^+^ extracellular vesicles was calculated as follows: [number of gp120/41-SA-Gold^+^ beads / (total number of beads)] x 100. The proportion of PD-L1^+^ extracellular vesicles with CD9 and/or CD63 and/or CD81 was calculated as follows: [number of gp120/41-SA-Gold^-^ and CD9^+^ and/or CD63^+^ and/or CD81^+^ beads / (total number of beads)] x 100.

### Characterization of HIV virions

HIV virions were characterized using mass cytometry based assay. Briefly, CBA Functional Beads (Becton Dickinson; #558578, #558656, #560038, #558582 and #558586) were conjugated with either anti-gp120/41 mAbs (clones 10–1074, PG9, VRC07, F240, 10E8) or an isotype-control (Biolegend; #401402) using the Functional Bead Conjugation Buffer Set protocol (BD; #558556) following the manufacturer’s instructions. Plasma were then incubated (2 hours, 4°C) with conjugated beads (9x10^4^ beads), washed (900g, 4 min) with PBS and incubated (30 min, 4°C) with isotope labelled mAbs directed to CD4, PD-L1, HLA-DR, α4β7 and an isotype-control and fixed (1 hour, RT) in 3.2% PFA buffer. Labeled samples were acquired on a Helios instrument (Fluidigm) using a flow rate of 0.030 ml/min. Data were analyzed using Fluidigm Cytobank software package (Cytobank, Mountain View, CA).

### Cell culture

Cells were cultured in RPMI (Gibco; Life Technologies) containing 10% heat-inactivated FBS (Institut de Biotechnologies Jacques Boy), 100 IU/ml penicillin and 100 μg/ml streptomycin (Bio Concept). PD-1/NFAT reporter Jurkat recombinant cell line (BPS Bioscience #60535) were cultured in DMEM (Life Technologies; #41965–039) containing 10% heat-inactivated FBS (Institut de Biotechnologies Jacques Boy), 100 IU/ml penicillin and 100 μg/ml streptomycin (Bio Concept), 1mg/ml Geneticin (Thermofisher; #10131027), and 200 μg/ml of Hygromycin B (Chemie brunschwig; #MED30-240-CR).

### *In vitro* production of soluble PD-L1 from HIV infected CD4 T cells and *in vitro* derived macrophages

Blood mononuclear cells were isolated from 3 HIV-uninfected individuals as previously described [83]. CD14-positive cells were isolated from PBMCs using CD14^+^ MicroBeads (Miltenyi; #130-050-201) as previously described [85]. CD14^+^ monocytes were differentiated on monocyte-derived macrophages in presence of GM-CSF (50 ng/mL; peprotech) for 6 days as previously described [86]. CD4 T cells were isolated from the CD14-negative fraction using EasySep Human CD4^+^ T Cell Isolation Kit (Stemcell; #17952) as previously described [23]. Purity of isolated cell populations was assessed by flow cytometry as previously described [23] and exceeded 95% in all experiments. CD4 T cells were stimulated (24 hours at 37°C) with 10 μg/ml anti-CD3 (BD; #555329) and anti-CD28 (BD; #555726) as previously described [87]. Monocyte-derived macrophages and activated CD4 T cells were exposed to HIV lab-derived variants (Bal-L, IIIB or JR-CSF; from NIH AIDS reagent program; #510, #398, #394) at 200 pg p24/million cells in presence or absence of emtricitabin (75 nM; from NIH AIDS reagent program #10071) or remained uninfected. Supernatants were collected at day 10 post infection and HIV-1 RNA levels were quantified using by COBAS AmpliPrep/TaqMan HIV-1 Test (Roche; Switzerland) as previously described [88]. Soluble PD-L1 levels were quantified by ELISA (*c*.*f*. quantification of soluble IC-Ls section). Cellular PD-L1 expression was assessed on activated CD4 T cells and monocyte-derived macrophages by mass cytometry as previously described [23].

### Immunocapture assay

PD-L1^+^ vesicles were characterized for their HIV RNA using a bead-based immuno-captured assay. Briefly, magnetic epoxy beads (Thermo-fischer; #14301) were conjugated with either anti-PD-L1 mAbs (MIH1, Invitrogen; #14-5983-82), anti-gp120/41 mAbs (10–1074, PG9, VRC07, F240, 10E8), Vedoluzymab (Takeda) or an isotype-control (Biolegend; #401402) following the manufacturer’s instructions. Plasma were then incubated (18 hours, 4°C) with conjugated beads (1x10^6^ beads), washed three times (900g, 4 min) with PBS 0.5% Tween and incubated (30 min, RT) with viral lysis buffer (Qiagen; #19073). Beads supernatants were collected and HIV-1 RNA levels were quantified using by COBAS AmpliPrep/TaqMan HIV-1 Test (Roche; Switzerland) as previously described [88]. The proportion of PD-L1^+^ vesicles with HIV RNA was calculated as follows: [HIV RNA (copies/mL) detected in anti-PD-L1 mAbs coated bead samples / (HIV RNA (copies/mL) detected in anti-gp120/41 coated beads)] x 100. The proportion of HIV RNA containing vesicles captured with isotype-control coated beads was calculated as follows: [HIV RNA (copies/mL) detected in isotype-control coated bead samples / (HIV RNA (copies/mL) detected in anti-gp120/41 mAbs coated beads)] x 100.

### *In vitro* HIV infection assay

Blood mononuclear cells were isolated from 7 HIV-uninfected individuals as previously described [83]. CD4 T cells were isolated from PBMCs using EasySep Human CD4^+^ T Cell Isolation Kit (Stemcell; #17952) as previously described [23]. Purity of isolated cell populations was assessed by flow cytometry and exceeded 95% in all experiments. CD4 T cells were stimulated (24 hours at 37°C) with 10 μg/ml anti-CD3 (BD; #555329) and anti-CD28 (BD; #555726) as previously described [88]. Magnetic epoxy beads (Thermo-fischer; #14301) were conjugated with either anti-PD-L1 mAbs (MIH1, Invitrogen; #14-5983-82), F240 (NIH AIDS reagent program; #7623), or an isotype-control (Biolegend; #401402) following the manufacturer’s instructions. Plasma of 7 viremic HIV-infected individuals were then incubated (18 hours, 4°C) with conjugated beads (1x10^6^ beads), and washed three times (900g, 4 min) with PBS 0.5% Tween. Beads were added to the stimulated CD4 T cells. Supernatants were collected at day 7 and day 14 post infection and HIV-1 RNA levels were quantified using by COBAS AmpliPrep/TaqMan HIV-1 Test (Roche; Switzerland). The proportion of HIV RNA levels in culture supernatants of cells infected with vesicles captured with anti-PD-L1 mAbs coated beads was calculated as follows: [HIV RNA (copies/mL) detected in anti-PD-L1 mAbs coated bead samples / (HIV RNA (copies/mL) detected in anti-gp41 (F240) mAbs coated beads)] x 100. The proportion of HIV RNA levels in culture supernatants of cells infected with vesicles captured with isotype-control coated beads was calculated as follows: [HIV RNA (copies/mL) detected in isotype-control coated bead samples / (HIV RNA (copies/mL) detected in anti-gp41 (F240) mAbs coated beads)] x 100.

### Preparation of PD-L1 lentiviruses

Viral particles were prepared by transfection of 293 T cells. 10–12 million 293 T cells were seeded per 15 cm tissue culture dishes in DMEM media with 10% FBS and Penicillin-Streptomicin, Gentamicin (50 μg/ml, GIBCO). The next day, cells were transfected with 70 μg of plasmid DNA (Rev, Gag/Pol, VSVG and PD-L1), through Calcium phosphate transfection (Takara) as previously described [89]. The next day, media were replaced with DMEM containing 5% FCS. After 2 days, media was filtered at 0.45 μM, collected into 38.5mL ultraclear centrifuge tubes (Herolab 253050), centrifuged at 50,000 g for 2 hours at 16°C. Virus pellet was re-suspended in 1 mL PBS, aliquoted and snap frozen in dry ice and then at -80°C for later use. Viral titers were determined by the assessment of HIV p24 antigen by ECL COBAS HIV Ag (Roche; Switzerland), HIV-1 RNA by COBAS AmpliPrep/TaqMan HIV-1 Test (Roche; Switzerland) and the percentage of GFP^+^ CD4 T cells with increasing virus doses at day 3 post-infection by flow cytometry.

### Production of PDL1^high^ and PD-L1_low_ HIV virions

Blood mononuclear cells were isolated from 3 HIV-uninfected individuals as previously described [83]. CD4 T cells were isolated from PBMCs using EasySep Human CD4^+^ T Cell Isolation Kit (Stemcell; #17952) as previously described [23]. Purity of isolated cell populations was assessed by flow cytometry and exceeded 95% in all experiments. CD4 T cells were stimulated (24 hours at 37°C) with 10 μg/ml anti-CD3 (BD; #555329) and anti-CD28 (BD; #555726) as previously described [88]. Activated CD4 T cells were transduced with GFP lentivirus (Addgene; #24129) or PD-L1/GFP dual reporter lentivirus (Addgene; #24129 with the inserted sequence of _fl_PD-L1; https://www.uniprot.org/uniprot/Q9NZQ7). After 48 hours, cells were washed and stained (4°C, 20 min) with LIVE/DEAD Fixable Aqua Dead Cell Stain Kit (Invitrogen; #L34957) and viable (Aqua-negative) GFP-positive and GFP-negative cells were sorted using BD Aria sorter. Sorted GFP-positive or GFP-negative cell populations were exposed to HIV lab-derived variants (Bal-L, IIIB or JR-CSF from NIH AIDS reagent program; #510, # 398, #394; 200 pg p24/million cells) or remained uninfected. Supernatants were collected at 14 days and virus concentrations were determined using HIV-1 RNA levels by COBAS AmpliPrep/TaqMan HIV-1 Test (Roche; Switzerland) as previously described [88]. Soluble PD-L1 levels were quantified by ELISA (*c*.*f*. quantification of soluble IC-Ls section). Supernatants containing HIV virions were stored at -80°C. The proportion of virions with PD-L1 were also assessed as previously described (*c*.*f*. Characterization of HIV virions section).

### Protein expression and purification

For PD-L1 transmembrane domain (amino acids: Thr239—Phe259) and intracytoplasmic tail (amino acids: Arg260- Thr 290) expression and purification. The cDNA encoding the tail and transmembrane domain of PD-L1 were synthetize with GST as fusion protein by Twist bioscience and cloned in pET-21(+) expression plasmid (Merck Millipore). Constructs were transformed into BL21Rosetta (DE3) E. coli cells (Novagen). Bacteria were grown in LB medium (Sigma) supplemented with 34 μg/L of chloramphenicol (Sigma) and 50 μg/L of ampicillin (Sigma) at 37°C, with shaking at 220 rpm, until an OD600 of 0.7 was reached. Protein expression was induced by addition of 1 mM isopropyl-thio-β-D-galactopyranoside (Sigma) and allowed to proceed for 4 h at 37°C, with shaking at 220 rpm, before cells were harvested by centrifugation. Cell pellets were suspended in extraction buffer (20 mM Tris-HCl, 0.5 M NaCl, 5 mM imidazole, 1mM 2-mercaptoethanol pH 8.0), homogenized and the obtained lysate was cleared by centrifugation and filtered through a 0.22 μm filter. The protein was purified from the filtered supernatant by IMAC via gravity column with cOmplete His-Tag Purification Resin (Roche) using extraction buffer as a wash and PBS supplemented with 1 M imidazole as elution. Peak fraction was concentrated in 5 kDa MWCO centrifugal filters, sterile filtered (0.22 μm) and applied to a PD10 buffer exchange column using PBS. The target proteins were snap frozen in liquid nitrogen and stored at -80°C.

### Biolayer interferometry experiment

FortéBio Octet K2 instrument was used to measure binding of recombinant _fl_PD-L1 (Amsbio; TP710226), _ΔC_PD-L1 (R&D system; 156-B7-100), intracytoplasmic domain (ic) and transmembrane domains (tm) or proteins to immobilized p17 or p24 HIV proteins. To immobilize p17 or p24 HIV proteins, Octet Aminopropylsilane Biosensors Biosensors (Sartorius) was pre-loaded at 800 nm for 15 minutes and saturated for 10 minutes in a solution containing BSA at 500 nm prior to be dipped in a new well. Biosensor tips were then equilibrated for 12 minutes prior to measuring association and dissociation for 8 minutes each with either recombinant _fl_PD-L1, or _ΔC_PD-L1, ic or tm proteins. BLI was also used for calculation of kinetic parameters in the same setting with BSA used as reference for baseline. Assays were performed with agitation set at 1,000 rpm in phosphate-buffered saline (PBS) supplemented with 0.01% BSA and 0.002% Tween-20. Data analysis and curve fitting (model 1.1) were carried out using Octet software, version 8.

### Characterization of HIV virions using transmission electron microscopy

Monocyte-derived macrophages were infected using HIV lab-derived variant (Bal-L #510 from NIH AIDS reagent program) at 200 pg p24/million cells. At 10 days post-infection, cells and supernatants were collected and fixed using 4% of formaldehyde and 0.1% glutaraldehyde (EMS, Hatfield, PA, US) in Phosphate Buffer (PB 0.1M pH7.4) (Sigma, St Louis, MO, US). The samples were then washed three times in PB buffer and spin down in low melting agarose 2% in H_2_O (Sigma, St Louis, MO, US), let to solidify on ice, cut in 1mm^3^ cube and dehydrated in ethanol solution (Sigma, St Louis, MO, US) at graded concentrations (30%-40min; 70%-40min; 100%-2x1h). This was followed by infiltration in LR White (Sigma, St Louis, MO, US) at graded concentrations (LR White 1/3 ethanol-2h; LR White 3/1 ethanol -2h; LR White 1/1-4h; LR White 1/1-12h) and finally polymerized for 24h at 50°C in oven under nitrogen gas. Ultrathin sections of 50nm were cut on a Leica Ultracut (Leica Mikrosysteme GmbH, *Vienna*, Austria) and picked up on a copper slot grid 2x1mm (EMS, Hatfield, PA, US) coated with a polystyrene film (Sigma, St Louis, MO, US). Sections were incubated with Glycine (50mM, RT, 5min) in PBS buffer (Sigma, St Louis, MO, US) followed by two incubations in BSAc 1% in PBS (Aurion, Wageningen, Netherlands) at RT for 5min. For the dual immunogold labelling, primary antibodies were diluted in PBS BSAc 0.1% and sections were incubated either with anti-p24 (1:100 dilution, Abcam; #ab63913) and biotinylated anti-PD-L1 (100μg/mL, eBioscience; #13-5983-82) or only anti-p24 (RT, 1 hour) for the single labelling. Sections were further washed 3 times with PBS BSAc 0.1% (RT, 5 min) and incubated with 15 nm Gold-labelled Goat-anti-Rabbit IgG (1:30 dilution in PBS BSAc 0.1%, Aurion; #815.011) and 6 nm Gold-labelled Streptavidin (1:30 dilution in PBS BSA 0.1%, Aurion; #806.099) (RT, 1hour). Sections were washed four times with PBS (RT, 5 min), and then incubated in glutaraldehyde 2.5% in H_2_O (RT, 2 min) and washed 10 times in H_2_O. Finally, sections were incubated in 2% uranyl acetate (Sigma, St Louis, MO, US) at RT for 10 min and washed several times in H_2_O. Micrographs were taken with a transmission electron microscope Philips CM100 (Thermo Fisher Scientific, Waltham, MA USA) at an acceleration voltage of 80kV with a TVIPS TemCam-F416 digital camera (**TVIPS GmbH, Gauting, Germany**).

### PD-1/NFAT cell reporter assay

PD-1/NFAT reporter Jurkat recombinant cell line (BPS Bioscience #60535) were incubated for 24 hours in 96-well flat-bottom plates (10’000 cells/per well in 200 μL of media) pre-coated with 1 μg/ml anti-CD3 (BD; #555329) and anti-CD28 (BD; #555726) (2h at 37°C). Cells were cultured in presence or in absence of *in vitro* produced immobilized PD-L1^high^ or PD-L1_low_ HIV virions (*c*.*f*. Production of PDL1^high^ and PD-L1_low_ HIV virions section), in the presence or the absence of anti-PD-L1/2 blocking mAbs (10 μg/mL; Ebioscience; # 16-5983-82, # 16-5888-82) for 24hours. To immobilize i*n vitro* produced HIV virions, wells were coated with non-neutralizing anti-gp41 mAbs (clone F240, 10 μg/mL; 4h at 37°C). PD-L1^high^ HIV virions were plated at increasing concentrations ranging from 22 to 180 pg p24/well in the presence or the absence of anti-PD-L1/2 blocking mAbs. Of note, all experiments were performed in presence of 75 nM emtricitabine to prevent infection. As control, recombinant PD-L1 (R&D Systems; #56-B7-100; 10 μg/mL) was coated (2h at 37°C). After 24hours, the luciferase activity was measured to monitor the impact of PDL1^high^ HIV virions using the ONE-Step Luciferase assay system as recommended by the manufacturer (BPS Bioscience; #60690). The relative light units were measured on a Synergy H1 instrument (BioTek) using the BioTek Gen5 v.3.0.3 Software.

### Influence of PD-L1^+^ virions on early TCR mediated signaling of primary CD4 T cells

CD4 T cells were isolated from cryopreserved mononuclear cells isolated from tonsils of 3 HIV-uninfected individuals using EasySep Human CD4+ T Cell Isolation Kit (Stemcell; #17952) as previously described [88]. Purity of isolated CD4 T cells was assessed by flow cytometry and exceeded 95% in all donors. Isolated CD4 T cells were then rested for 2 hours in RPMI (Gibco; Life Technologies) containing 2% heat-inactivated FBS (Institut de Biotechnologies Jacques Boy), 100 IU/ml penicillin and 100 μg/ml streptomycin (Bio Concept). Cells (2 x 10^5^) were then stimulated (5 minutes; 37°C) or not in 96-well plates coated (2 hours; 37°C) with 10 μg/ml anti-CD3 (BD; #555329) and anti-CD28 (BD; #555726) in presence or in absence of *in vitro* produced immobilized PD-L1^high^ or PD-L1_low_ HIV virions (*c*.*f*. Production of PDL1^high^ and PD-L1_low_ HIV virions section). To immobilize i*n vitro* produced HIV virions, wells were coated with non-neutralizing anti-gp41 mAbs (clone F240, 10 μg/mL; 4h at 37°C). Wells were then exposed to *in vitro* produced PD-L1^high^ or PD-L1_low_ HIV virions (90 pg p24/well; 2 hours; 37°C). Wells were then washed twice with PBS. Plated cells were then centrifuged for 60 seconds at 250-RPM to help synchronize the contact of cells with the TCR activating surface. The cellular phosphorylation states were quenched with 2% PFA after 5 minutes stimulation or at T = 0. Fixed cells were washed (RPMI 2% FBS) and multiplexed (30 min; 4°C) with anti-CD45 antibody coupled with metal isotopes Y-89, Pr-141, Pt-194, Pt-195, Pt-196 or Pt-198. Stained cells were washed once (RPMI 10% FBS), pooled and treated with 1.4-fold volume of Smart tube stabilizer (Invitrogen; # 501351692) according to the manufactures protocol before sample storage (-80°C). Multiplexed samples were then thawed, washed (RPMI 10% FBS) and stained using a panel of metal conjugated antibodies targeting CD3, CD4, CD8, CD45RA, CD45RO, PD-1, phospho-ZAP70 and phospho-SLP76. Samples were analyzed on a Helios mass cytometer with data analysis performed using Cytobank analysis software. Multiplexed samples were debarcoded with the anti-CD45 metal isotope labels as previously described [90] and PD-1^-^ and PD-1^+^ CD45RO^+^ CD4 T cells were evaluated for mean intensity increases and percentages of phospho-ZAP70 and phospho-SLP76 levels relative to the unstimulated conditions.

### T follicular helper cell proliferation assay

Cryopreserved mononuclear cells isolated from tonsils of 10 HIV-uninfected individuals were thawed, stained with Aqua LIVE/DEAD stain kit (4°C; 15 min), washed and stained (4°C; 30 min) with conjugated mAbs to CD4, CD19, IgD, CD38 and PD-1. Viable Tfh cells (CD4^+^PD-1^+^) and GC B cells (CD19^+^CD38^+^IgD^-^) were sorted using FACSAria (Beckton & Dickinson) as previously described [21]. In all sorting experiments the grade of purity of the sorted cell populations was >98%. Isolated Tfh cells were stained with 0.25μM 5,6-carboxyfluorescein, succinimidyl ester (CFSE, Molecular Probes, USA) as previously described [91]. CFSE labelled Tfh cells co-cultured with autologous GC B cells (ratio 1:1) and stimulated (6 days; 37°C) or not with Staphyloccocus enterotoxin B (SEB; 250 ng/mL) in presence or in absence of *in vitro* produced immobilized PD-L1^high^ or PD-L1_low_ HIV virions (*c*.*f*. Production of PDL1^high^ and PD-L1_low_ HIV virions section), in the presence or in the absence of anti-PD-L1/2 blocking mAbs (10 μg/mL; Ebioscience; # 16-5983-82, # 16-5888-82). To immobilize i*n vitro* produced HIV virions, wells were coated with non-neutralizing anti-gp41 mAbs (clone F240, 10 μg/mL; 4h at 37°C). Wells were then exposed to *in vitro* produced PD-L1^high^ or PD-L1_low_ HIV virions (90 pg p24/well; 2 hours; 37°C). Wells were washed with twice with PBS. At day 6, cells were harvested, stained (4°C; 20 min) using aqua LIVE/DEA D stain kit, washed (400 g; 5 min) and stained (4°C; 30 min) with mAbs to CD3, CD4, CD8. Frequencies of proliferating CFSElow Tfh cells were assessed by flow cytometry.

### Flow cytometry analyses

Cells were fixed with CellFix (#340181; BD), acquired on an LSRII SORP (4 lasers: 405, 488, 532 and 633 nm) and analyzed using FlowJo (version 10.6.2) (Tree star Inc, Ashland, OR, USA) and SPICE version 6.0 downloaded from http://exon.niaid.nih.gov/spice [92].

### Statistical analyses

Statistical significance (*P* values) was obtained using one-way ANOVA (Kruskal-Wallis test or Friedman Test) followed by a Dunn’s multiple comparison test, by using a Mann-Whitney test, or by using Spearman’s rank correlations using GraphPad Prism version 9.1.0 (San Diego, CA).

## Supporting information

S1 Fig Schematic representation and validation of PD-L1^+^ vesicles capture assay. **A.** Schematic representation of PD-L1^+^ vesicles capture assay. Beads were coated with anti-PD-L1 mAbs and incubated with plasma to capture PD-L1^+^ vesicles. Immunocaptured PD-L1^+^ vesicles were labelled with isotope labelled anti-gp120/41 cocktail mAbs (markers of HIV virions) and isotope-labelled anti-CD9, anti-CD63 and anti-CD81 (markers of exosomes). Beads coated with an isotype-control were also used. **B**. Representative mass cytometry profiles of vesicles captured with isotype-control coated beads of plasma from one HIV-uninfected individual (HS#011) and one viremic HIV-infected individual (HIV#0615). **C**. Cumulative data showing the proportion of PD-L1^+^ vesicles captured either with isotope coated beads or anti-PD-L1 mAbs coated beads of plasma from HIV-uninfected individuals (N = 10) and viremic HIV-infected individuals (N = 20). Histograms correspond to the mean and red error bars correspond to the SEM (**C**). Black stars indicate statistical significance (** = *P*<0.01; **** = *P*<0.0001). Statistical significance (*P* values) was obtained using one-way ANOVA (Kruskal-Wallis test) followed by either Wilcoxon matched-pairs signed rank test for paired comparisons or by a Mann-Whitney test for unpaired comparison **(C)**.
(TIF)

S2 Fig Proportion of PD-L1^+^ vesicles with gp120/41 and/or CD9/CD63/CD81 captured from plasma of viremic HIV-infected individuals collected during the acute phase (N = 10) or during the chronic phase (N = 10). Histograms correspond to the mean and red error bars correspond to the SEM. n.s. indicate no statistical significance (*P>*0.05). Statistical significance (*P* values) was obtained using one-way ANOVA (Kruskal-Wallis test) followed by a Mann-Whitney test.
(TIF)

S3 Fig Schematic representation of gp120/41^+^ vesicles capture assay. Beads were coated with anti-gp120/41 cocktail mAbs and incubated with plasma to capture gp120/41^+^ vesicles. Immuno-captured HIV virions were labelled with biotinylated antigp120/41 cocktail mAbs and isotope-labelled anti-α4β7, anti-HLA-DR, anti-CD4 and anti-PDL1 mAbs. Streptavidin-gold was used to detect biotinylated anti-gp120/41 cocktail mAbs. Beads coated with an isotype-control were also used.
(TIF)

S4 Fig HIV RNA levels detected in culture supernatants of HIV-infected MDM and activated CD4 T cells. MDM and activated CD4 T cells from HIV-uninfected individuals (N = 4) were infected with HIV-lab-derived variants and cultured for 14 days in absence of emtricitabin. HIV RNA levels were assessed in culture supernatants at day 14 post infection. Some experiments were conducted with three distinct HIV lab-derived variants (Bal, IIIB and JR-CSF). Histograms correspond to the mean and red error bars correspond to the SEM. n.s. indicate no statistical significance (*P>*0.05). Statistical significance (*P* values) was obtained using one-way ANOVA (Kruskal-Wallis test) followed by a Dunn’s multiple comparison test.
(TIF)

S5 Fig Influence of PD-L1^+^ virions on early TCR mediated signaling of primary CD4 T cells. CD4 T cells isolated from tonsils of HIV-uninfected individuals (N = 3) were stimulated with anti-CD3/CD28 mAbs for 5 minutes in presence or in absence of *in vitro* produced PD-L1_low_ HIV virions or PD-L1^high^ HIV virions. As controls, cells remained unstimulated. The phosphorylation of ZAP70 and SLP76 was assessed by mass cytometry on PD-1^-^ and PD-1^+^ memory (CD45RO^+^) CD4 T cells and used as markers of early TCR signaling cascade. **A**. Gating strategy. **B**. Heatmap representing the mean signal intensity of phospho-ZAP70 and phospho-SLP76 signaling proteins of TCR stimulated PD-1^-^ and PD-1^+^ memory CD4 T cells in presence or in absence of PD-L1_low_ or PD-L1^high^ HIV virions as compared to unstimulated condition of one representative subject (#111A). The change of color (blue: negative; yellow: positive) indicate the mean signal intensity of phosphorylated signaling proteins. Cumulative data representing the percentage of phopho-ZAP70 (**C**) or phospho-SLP76 (**D**) of PD-1^-^ and PD-1^+^ CD4 T cells stimulated or not in presence or in absence of PD-L1_low_ or PD-L1^high^ HIV virions (N = 3). Histograms correspond to the mean and black error bars correspond to the SEM. Statistical significance (*P* values) was obtained using one-way ANOVA (Kruskal-Wallis test) followed by a Dunn’s multiple comparison test.
(TIF)
