# Supplementary figures and images for: Active PD-L1 incorporation within HIV virions functionally impairs T follicular helper cells
Source: PLoS Pathog. 2022 Jul 5;18(7):e1010673. doi: 10.1371/journal.ppat.1010673 (PMC9286290; doi:10.1371/journal.ppat.1010673)

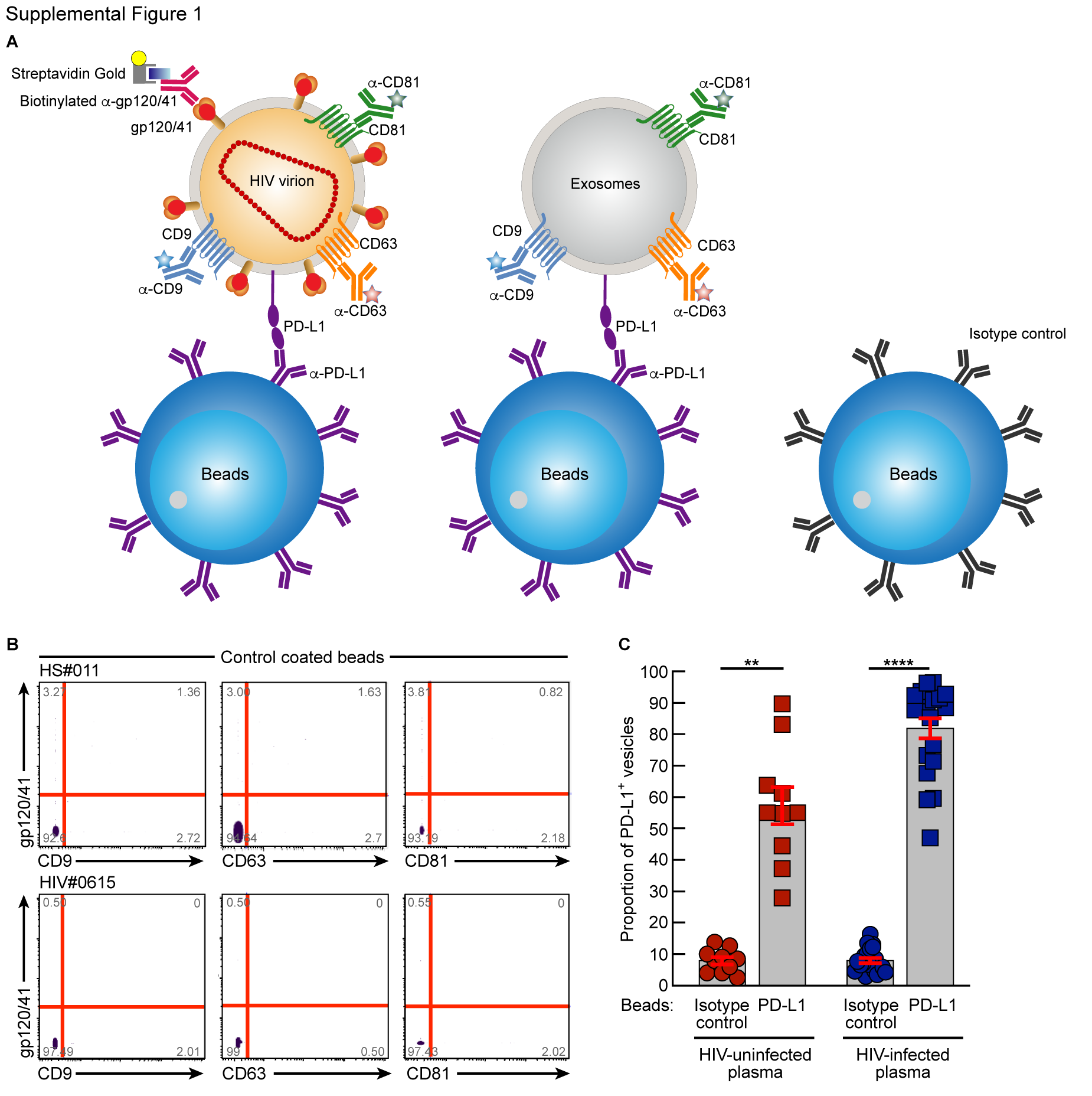

Supplement: S1 Fig — A. Schematic representation of PD-L1+ vesicles capture assay. Beads were coated with anti-PD-L1 mAbs and incubated with plasma to capture PD-L1+ vesicles. Immunocaptured PD-L1+ vesicles were labelled with isotope labelled anti-gp120/41 cocktail mAbs (markers of HIV virions) and isotope-labelled anti-CD9, anti-CD63 and anti-CD81 (markers of exosomes). Beads coated with an isotype-control were also used. B. Representative mass cytometry profiles of vesicles captured with isotype-control coated beads of plasma from one HIV-uninfected individual (HS#011) and one viremic HIV-infected individual (HIV#0615). C. Cumulative data showing the proportion of PD-L1+ vesicles captured either with isotope coated beads or anti-PD-L1 mAbs coated beads of plasma from HIV-uninfected individuals (N = 10) and viremic HIV-infected individuals (N = 20). Histograms correspond to the mean and red error bars correspond to the SEM (C). Black stars indicate statistical significance (** = P<0.01; **** = P<0.0001). Statistical significance (P values) was obtained using one-way ANOVA (Kruskal-Wallis test) followed by either Wilcoxon matched-pairs signed rank test for paired comparisons or by a Mann-Whitney test for unpaired comparison (C). (TIF) [file ppat.1010673.s001.tif]

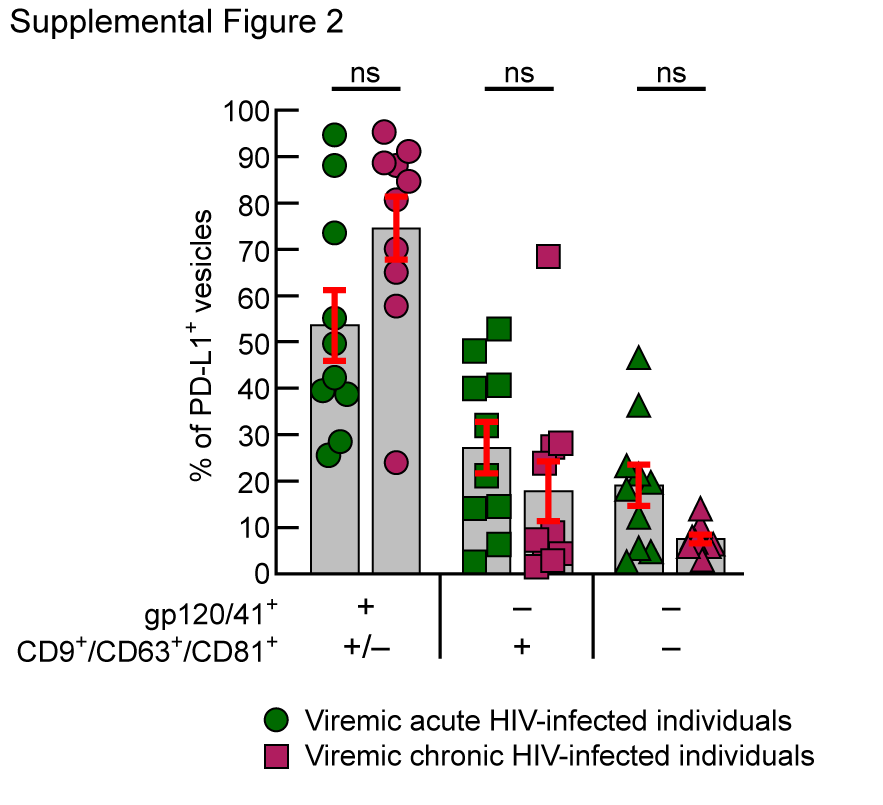

Supplement: S2 Fig — Histograms correspond to the mean and red error bars correspond to the SEM. n.s. indicate no statistical significance (P>0.05). Statistical significance (P values) was obtained using one-way ANOVA (Kruskal-Wallis test) followed by a Mann-Whitney test. (TIF) [file ppat.1010673.s002.tif]

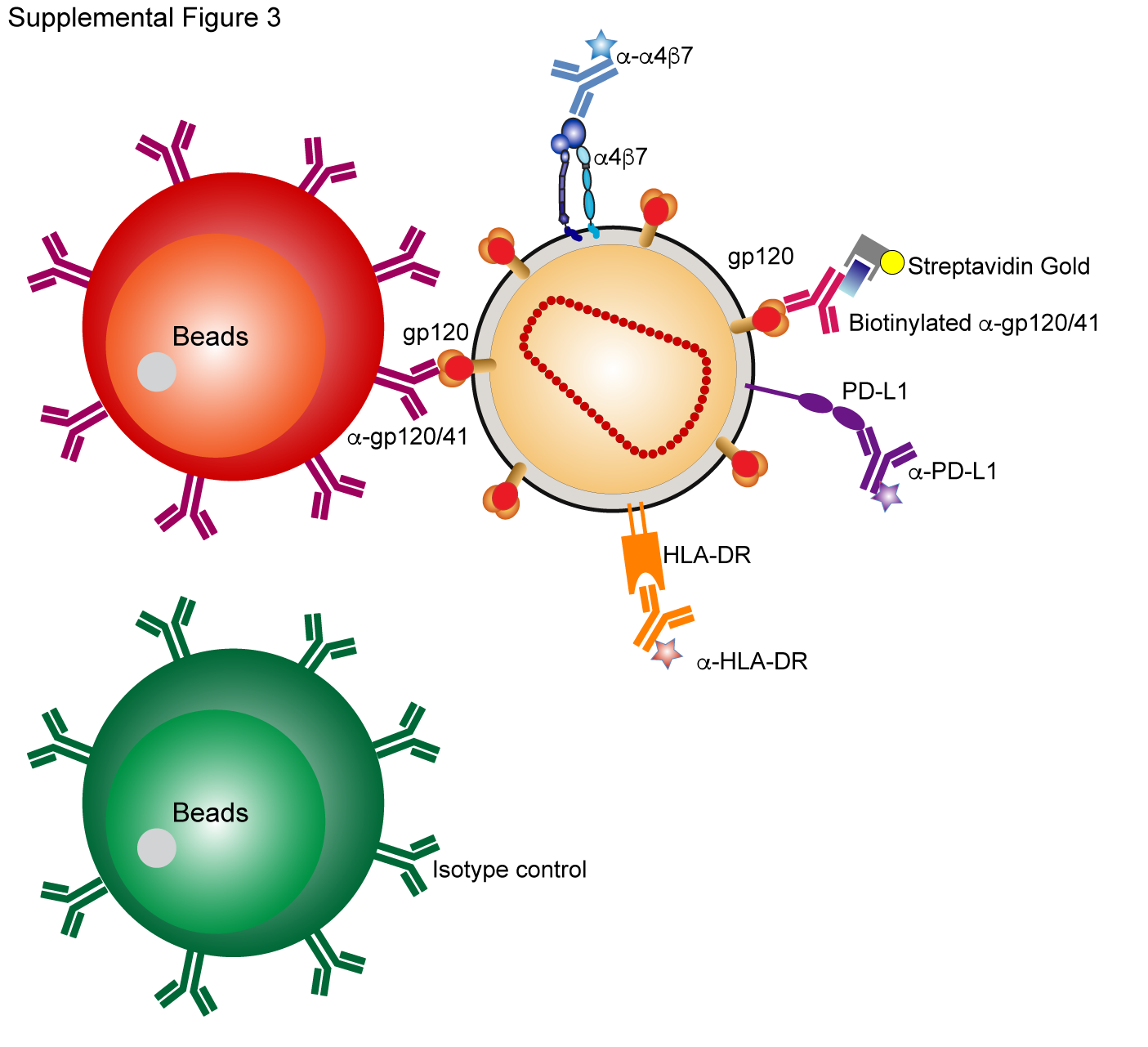

Supplement: S3 Fig — Beads were coated with anti-gp120/41 cocktail mAbs and incubated with plasma to capture gp120/41+ vesicles. Immuno-captured HIV virions were labelled with biotinylated antigp120/41 cocktail mAbs and isotope-labelled anti-α4β7, anti-HLA-DR, anti-CD4 and anti-PDL1 mAbs. Streptavidin-gold was used to detect biotinylated anti-gp120/41 cocktail mAbs. Beads coated with an isotype-control were also used. (TIF) [file ppat.1010673.s003.tif]

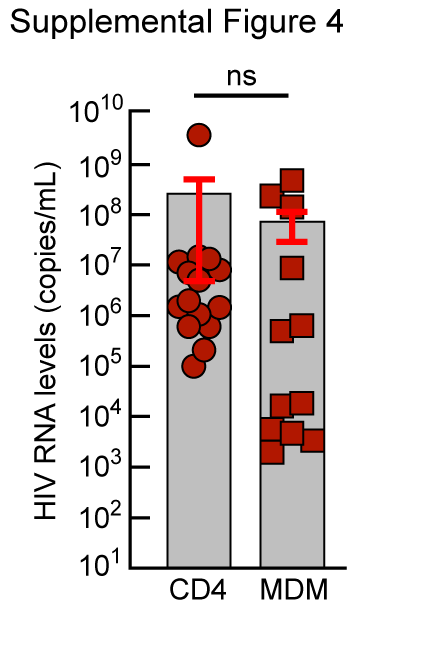

Supplement: S4 Fig — MDM and activated CD4 T cells from HIV-uninfected individuals (N = 4) were infected with HIV-lab-derived variants and cultured for 14 days in absence of emtricitabin. HIV RNA levels were assessed in culture supernatants at day 14 post infection. Some experiments were conducted with three distinct HIV lab-derived variants (Bal, IIIB and JR-CSF). Histograms correspond to the mean and red error bars correspond to the SEM. n.s. indicate no statistical significance (P>0.05). Statistical significance (P values) was obtained using one-way ANOVA (Kruskal-Wallis test) followed by a Dunn’s multiple comparison test. (TIF) [file ppat.1010673.s004.tif]

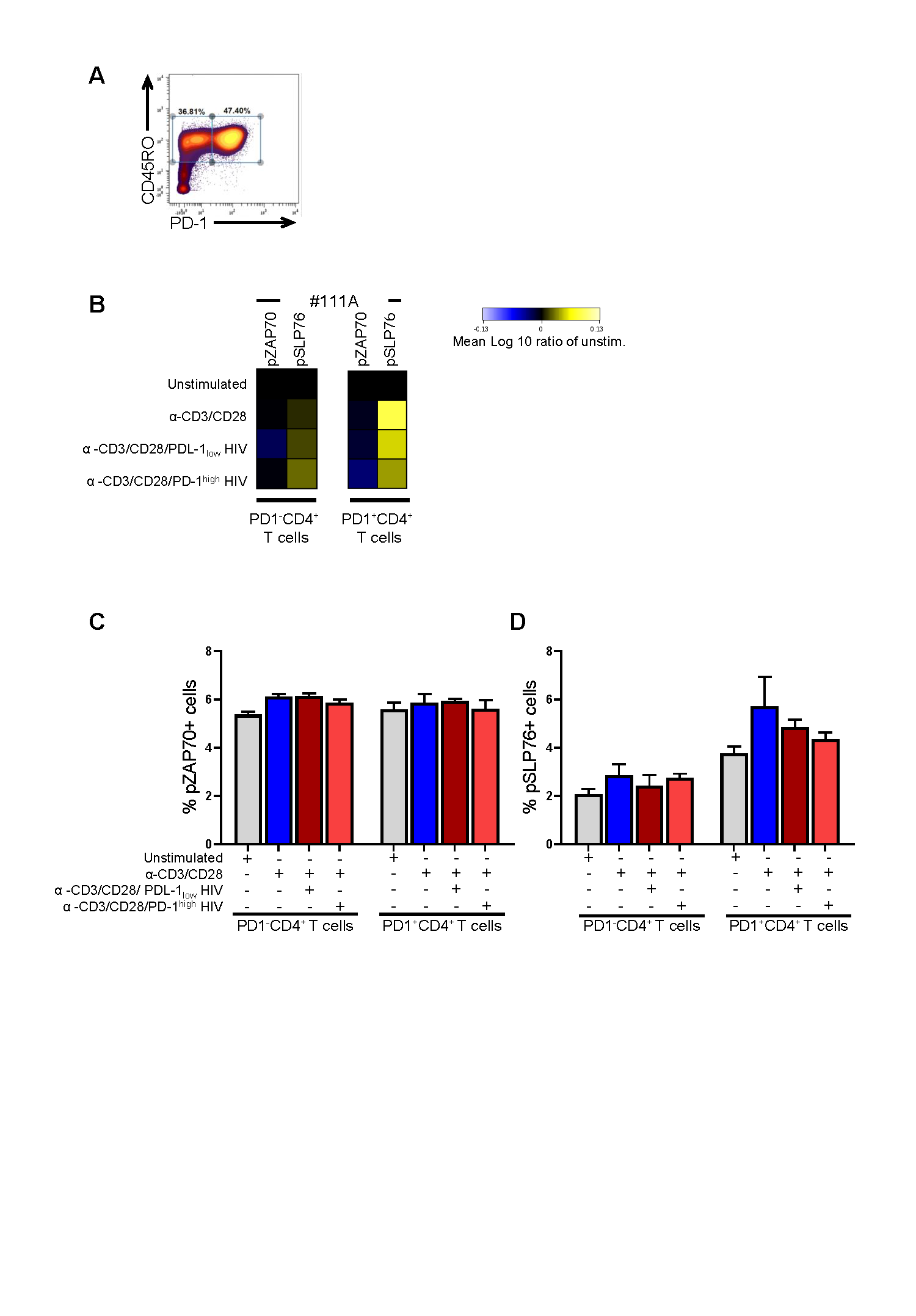

Supplement: S5 Fig — CD4 T cells isolated from tonsils of HIV-uninfected individuals (N = 3) were stimulated with anti-CD3/CD28 mAbs for 5 minutes in presence or in absence of in vitro produced PD-L1low HIV virions or PD-L1high HIV virions. As controls, cells remained unstimulated. The phosphorylation of ZAP70 and SLP76 was assessed by mass cytometry on PD-1- and PD-1+ memory (CD45RO+) CD4 T cells and used as markers of early TCR signaling cascade. A. Gating strategy. B. Heatmap representing the mean signal intensity of phospho-ZAP70 and phospho-SLP76 signaling proteins of TCR stimulated PD-1- and PD-1+ memory CD4 T cells in presence or in absence of PD-L1low or PD-L1high HIV virions as compared to unstimulated condition of one representative subject (#111A). The change of color (blue: negative; yellow: positive) indicate the mean signal intensity of phosphorylated signaling proteins. Cumulative data representing the percentage of phopho-ZAP70 (C) or phospho-SLP76 (D) of PD-1- and PD-1+ CD4 T cells stimulated or not in presence or in absence of PD-L1low or PD-L1high HIV virions (N = 3). Histograms correspond to the mean and black error bars correspond to the SEM. Statistical significance (P values) was obtained using one-way ANOVA (Kruskal-Wallis test) followed by a Dunn’s multiple comparison test. (TIF) [file ppat.1010673.s005.tif]
